# A Cascade Strategy Enables a Total Synthesis of (±)‐Morphine

**DOI:** 10.1002/anie.201608526

**Published:** 2016-10-13

**Authors:** Shuyu Chu, Niels Münster, Tudor Balan, Martin D. Smith

**Affiliations:** ^1^ Chemistry Research LaboratoryUniversity of Oxford12 Mansfield RoadOxfordOX1 3TAUK

**Keywords:** cascade reactions, metathesis, morphine, photochemistry, total synthesis

## Abstract

Morphine has been a target for synthetic chemists since Robinson proposed its correct structure in 1925, resulting in a large number of total syntheses of morphine alkaloids. Here we report a total synthesis of (±)‐morphine that employs two key strategic cyclizations: 1) a diastereoselective light‐mediated cyclization of an O‐arylated butyrolactone to form a tricyclic cis‐fused benzofuran and 2) a cascade ene–yne–ene ring closing metathesis to forge the tetracyclic morphine core. This approach enables a short and stereoselective synthesis of morphine in an overall yield of 6.6 %.

Morphine (**1**), named by Sertürner for its tendency to induce sleep, is the most abundant alkaloid isolated from the opium poppy *Papaver somniferum*.^1^ As the main constituent of opium, morphine's pain relieving properties have been exploited for thousands of years, and in modern times have resulted in its place on the World Health Organization's list of essential medicines.^2^ Its correct structure was proposed by Robinson in 1925,^3^ and was confirmed by Gates (through total synthesis, 1952)^4^ and Hodgkin (using X‐ray crystallography, 1955).^5^ Currently, all medicinal (and illicit) morphine is derived from natural sources; there is no synthetic route that competes on scale and cost with isolation from natural sources. This problem has been elegantly outlined in a recent perspective from Hudlicky, which focuses on his group's chemoenzymatic approaches to the morphine skeleton,^6^ and in other reviews which focus on the range of strategies employed in total syntheses of morphine alkaloids.^7^ Nonetheless, the combination of potent biological effects and a complex pentacyclic structure bearing five contiguous stereocentres has made morphine and its associated alkaloids an enduring challenge for synthetic chemistry, resulting in more than 30 total or formal syntheses of morphine alkaloids.^8^, ^9^ Our proposed strategy for the total synthesis of morphine is outlined below (Scheme 1). It has been demonstrated by Fuchs that the piperidine ring in morphine (**1**) can be made by an intramolecular 1,6‐addition of an amine analogous to that in **2**.^10^ This requires an α,β,γ,δ‐unsaturated ketone, which we envisaged could be constructed via a cascade ene–yne–ene ring closing metathesis from the heavily functionalized benzofuran **3**.^11^, ^12^ A metathesis cascade cyclization offers the potential for a new end‐game to the total synthesis of morphine in which the requisite functional groups are installed prior to the formation of the B and C rings. This has the advantage of minimizing late‐stage functional group interconversions once the ABCD tetracycle is complete. The benzofuran **3** could be synthesized from the tricyclic butyrolactone **4** by a series of functional group interconversions to install the vinyl ketone and alkyne functional groups. We recognized that the *cis*‐fused butyrolactone **4**, bearing an all‐carbon quaternary stereocentre, could be generated via a photocyclization from substituted butenolide **5**, with the relative stereochemistry a consequence of the 5,5‐*cis*‐fused nature of the tricycle. The butenolide **5** should be accessible through the connection of three components: phenol **6**, mucobromic acid (**7**) and protected amino‐borane **8**.

**Scheme 1:**
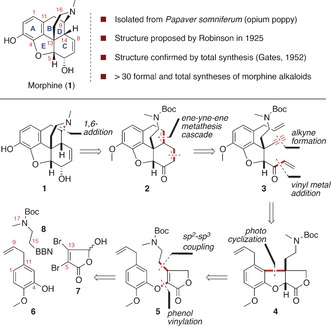
Strategy for the synthesis of (±)‐morphine.

Our route begins with the union of phenol **6** 13 with mucobromic acid (**7**) (Scheme 2). Treatment of phenol **6** with an excess of mucobromic acid (**7**) in the presence of aqueous sodium hydroxide led to smooth 1,4‐addition‐elimination with exclusive displacement of the bromide β to the masked aldehyde. In situ reduction of the resultant aldehyde and citric acid mediated lactonization afforded butyrolactone **9** in 86 % overall yield.^14^ The vinyl bromide in **9** can be cross‐coupled with protected β‐amino borane derivative **8** (synthesized by addition of 9‐borabicyclo[3.3.1]nonane across *tert*‐butyl methyl(vinyl) carbamate)^15^ by employing a Suzuki–Miyaura coupling. This sp^3^–sp^2^ coupling^16^ was particularly challenging and required extensive experimentation to discover the optimum conditions of catalytic Pd(dppf)Cl_2_⋅CH_2_Cl_2_ in the presence of cesium carbonate in an aqueous DMF/THF mixture at 40 °C. This yielded the required functionalized butenolide **5** in 58 % yield (on a greater than 5 g scale). We investigated the photocyclization of this material through irradiation with a high‐pressure mercury vapor lamp. In seminal work in this field, Schultz demonstrated that related substrates can cyclize efficiently via a process best described as a 6π photoelectrocyclization followed by a concerted 1,4‐hydrogen shift.^17^ After extensive investigation we were able to demonstrate that a mixed dichloroethane/hexafluoroisopropanol solvent system was optimal, leading to the formation of **4** in an isolated yield of 37 % after 120 h of irradiation (plus 22 % recovered starting material; yield 47 % based on recovered starting material, on a >2 g scale). As expected, only the *cis*‐fused 5,5‐system was isolated, presumably due to strain associated with the alternative *trans*‐fused stereochemistry. With this material in hand, we investigated the installation of a C‐14 alkyne, which requires a change in oxidation state at C‐14. This was achieved through ring opening of the lactone in **4** under basic conditions using aqueous KOH in *tert*‐butanol to afford a hydroxy acid, which was selectively oxidized using aqueous sodium ruthenate^18^ to afford hemiacetal **10**. This was difficult to isolate cleanly, and in practice was trapped without delay using dimethyl(diazomethyl)phosphonate in methanol in the presence of potassium carbonate to install the requisite C‐14 alkyne. This carboxylic acid intermediate was also challenging to isolate effectively and hence we employed 4‐(4,6‐dimethoxy‐1,3,5‐triazin‐2‐yl)‐4‐methylmorpholinium chloride to generate an activated ester in situ that was intercepted by *N*,*O*‐dimethylhydroxylamine in the presence of *N*‐methylmorpholine to afford Weinreb amide **11** in 73 % yield over two steps from **4**.^19^ Addition of vinylmagnesium bromide in THF at 0 °C proceeded smoothly to afford ketone **3** in 87 % yield. This heavily functionalized benzofuran is the key intermediate for our cascade approach to the morphine skeleton. Treatment of this intermediate with 5 mol % of the Hoveyda–Grubbs second generation catalyst^20^ in dichloromethane at room temperature afforded tetracycle **2** which could be isolated in 94 % yield. It is likely that this transformation proceeds via reaction of the ruthenium alkylidene precatalyst with the allyl component of **3**, followed by intramolecular reaction with the alkyne to ultimately give alkylidene intermediate **12**.^21^ Ring closing metathesis with the α,β‐unsaturated ketone affords the tetracycle **2** as effectively the sole product in this transformation.^22^ In practice this step could be combined with a subsequent 1,6‐addition through removal of the *tert*‐butoxycarbonyl group with trifluoroacetic acid to afford an *N*‐methyl ammonium salt followed by free‐basing with sodium carbonate to trigger the carbon–nitrogen bond formation. This affords a 10:1 mixture of neopinone **13** 23 and codeinone **14** that can be transformed into codeinone **14** by treatment with HCl in a mixture of dichloromethane and ether, using the method employed by Rapoport.^24^ This reaction likely proceeds via transient formation of a β‐halo ketone followed by elimination on treatment with aqueous sodium hydroxide to afford the thermodynamically favoured product **14**. This material can be isolated, but diastereoselective reduction of the C‐6 ketone can be achieved by the addition of sodium borohydride to afford codeine (**15**) as a single diastereoisomer in 65 % overall yield from **3**.^25^ Demethylation of the C‐3 methyl ether with boron tribromide afforded morphine **1** in 86 % yield.^26^ The ^1^H and ^13^C NMR spectra of our synthetic material were identical to those reported for both natural and other synthetic materials.

**Scheme 2:**
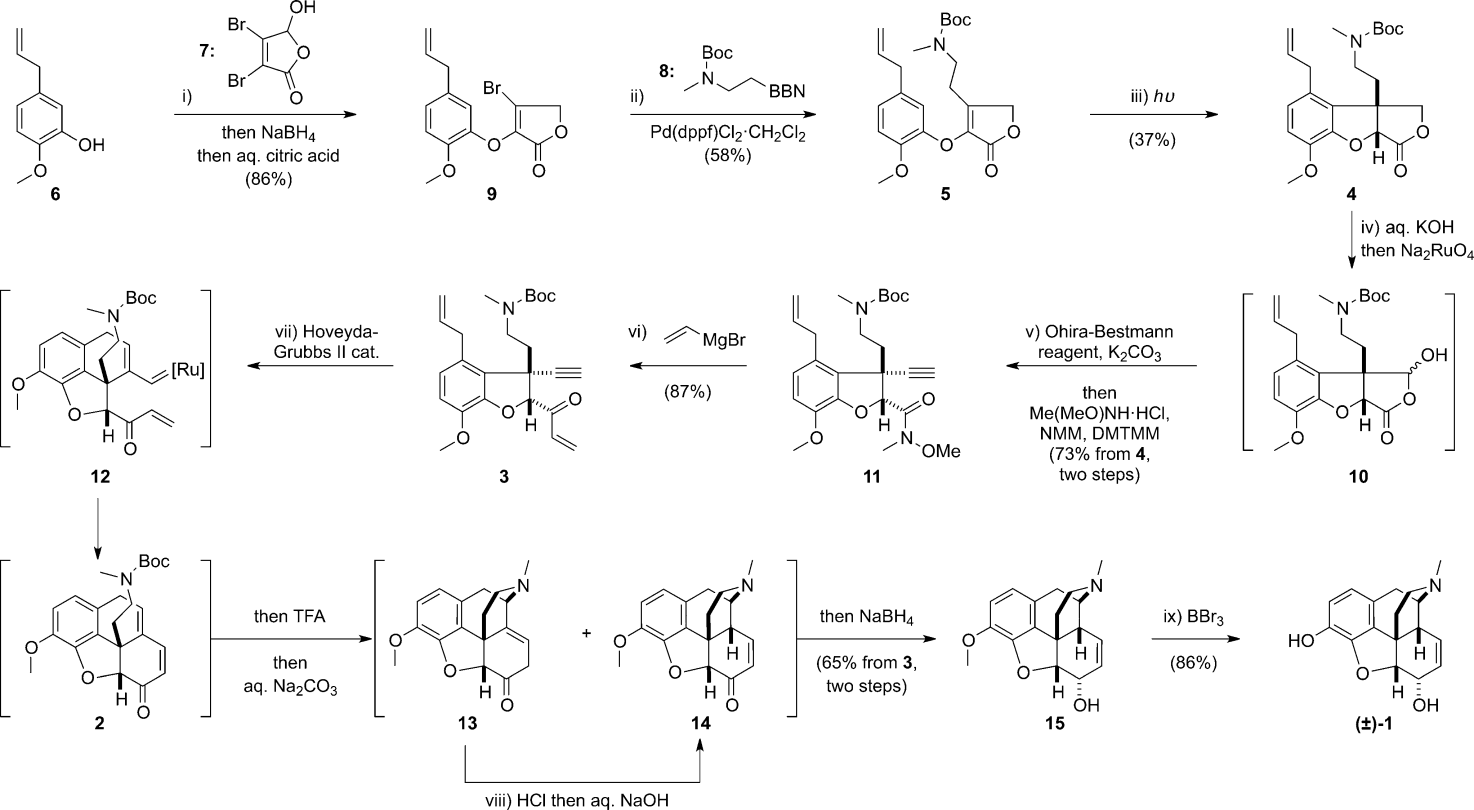
Total synthesis of morphine: i) Mucobromic acid **7** (5.0 equiv), aq. NaOH (9.5 equiv), 20 °C; then NaBH_4_ (4.5 equiv), 0 °C; then aq. citric acid (14 equiv), CHCl_3_/H_2_O, 20 °C. ii) *B*‐alkyl‐9‐BBN reagent **8** (1.2 equiv), Pd(dppf)Cl_2_⋅CH_2_Cl_2_ (0.1 equiv), Cs_2_CO_3_ (2.0 equiv), DMF/THF/H_2_O, 40 °C. iii) High‐pressure mercury vapor lamp irradiation with Pyrex filter, DCE/HFIP, 20 °C. iv) Aq. KOH (20 equiv), *t*‐BuOH/H_2_O, 20 °C; then aq. Na_2_RuO_4_ (1.1 equiv), 50 °C. v) Dimethyl(diazomethyl)phosphonate (2.0 equiv), K_2_CO_3_ (5.0 equiv), MeOH, 20 °C; then Me(MeO)NH⋅HCl (6.0 equiv), NMM (6.0 equiv), DMTMM (4.0 equiv), 20 °C. vi) Vinylmagnesium bromide (4.0 equiv), THF, 0 °C. vii) Hoveyda–Grubbs 2^nd^ generation catalyst (0.05 equiv), CH_2_Cl_2_, 20 °C; then TFA (40 equiv), 20 °C; then aq. Na_2_CO_3_ (60 equiv), 20 °C. viii) HCl (4.0 equiv), Et_2_O/CH_2_Cl_2_, 20 °C; then aq. NaOH (8.0 equiv), 20 °C; then NaBH_4_ (10 equiv), 20 °C. ix) BBr_3_ (6.0 equiv), CH_2_Cl_2_/CHCl_3_, 20 °C. BBN=9‐borabicyclo[3.3.1]nonane, dppf=1,1′‐ferrocenediyl‐bis(diphenylphosphine), DMF=dimethylformamide, THF=tetrahydrofuran, DCE=1,2‐dichloroethane, HFIP=hexafluoroisopropanol, NMM=4‐methylmorpholine, DMTMM=4‐(4,6‐dimethoxy‐1,3,5‐triazin‐2‐yl)‐4‐methylmorpholinium chloride, TFA=trifluoroacetic acid.

In conclusion, we have described a short^27^ and stereoselective synthesis of morphine in which a cascade ene–yne–ene metathesis provides a new disconnection of this most enduring of targets. The value of this approach is most clearly reflected in the installation of key functional groups at an appropriate oxidation level, which minimizes late stage transformations and protecting group manipulations. Our future work will focus on an enantioselective synthesis and further investigation of the photocyclization, which we hope may result in a more efficient and scalable synthetic route.

## Supporting information

As a service to our authors and readers, this journal provides supporting information supplied by the authors. Such materials are peer reviewed and may be re‐organized for online delivery, but are not copy‐edited or typeset. Technical support issues arising from supporting information (other than missing files) should be addressed to the authors.

Supplementary
