# Supplementary material for: A Cascade Strategy Enables a Total Synthesis of (±)‐Morphine
Source: Angew Chem Int Ed Engl. 2016 Oct 13;55(46):14306–9. doi: 10.1002/anie.201608526 (PMC5129523; doi:10.1002/anie.201608526)

Angewandte Chemie
Eine Zeitschrift der Gesellschaft Deutscher Chemiker

Supporting Information

A Cascade Strategy Enables a Total Synthesis of (±)-Morphine

*Shuyu Chu, Niels Münster, Tudor Balan, and Martin D. Smith**

anie_201608526_sm_miscellaneous_information.pdf

Table of Contents

General Information: ……………………………………………………………………………S2

Experimental Procedures: ……………………………………………………………………S3

Optimisation of the *B*-alkyl Suzuki-coupling: ……………………………………………S12

NMR spectra of intermediates and products: ………………………………………………S13

General Information

Reactions requiring moisture-sensitive reagents were carried out in oven-dried glassware, under an atmosphere of argon (balloon pressure). Dichloromethane (DCM), acetonitrile (MeCN), methanol (MeOH), chloroform ($CHCl_3$), and tetrahydrofuran (THF) were purified by filtration through activated alumina columns employing the method of Grubbs *et al.*[i] Water was purified by an Elix® UV-10 system. Reagents were used directly as supplied by major chemical suppliers, or following purification procedures described by Perrin and Armarego.[ii] Petroleum ether (PE) refers to the fraction of petroleum ether which boils in the range 40-60 °C unless otherwise stated. Brine refers to a saturated aqueous solution of sodium chloride.

Silica gel chromatography was carried out using Merck Geduran® Silicagel (40-63 μm particle size). Analytical thin layer chromatography was carried out using pre-coated, aluminum backed plates (Merck Kieselgel 60 F254). Visualization was achieved with ultraviolet irradiation (254 nm) and staining with basic permanganate.

NMR spectroscopy was carried out using Bruker Avance spectrometers in the deuterated solvent stated, using the residual non-deuterated solvent signal as an internal reference (^{1}H NMR: $CDCl_3$ 7.26 ppm, toluene-d_8 7.09, 7.01, 6.97, 2.08 ppm; ^{13}C NMR: $CDCl_3$ 77.16 ppm, toluene-d_8 137.48, 128.87, 127.96, 125.13, 20.43 ppm). Chemical shifts are quoted in ppm, based on appearance rather than interpretation. Signal patterns are indicated as: br, broad; s, singlet; d, doublet; t, triplet; q, quartet; m, multiplet.

Infrared spectra were prepared as a neat film and were recorded using a Bruker Tensor 27 FTIR spectrometer using an ATR module.

Low-resolution mass spectroscopy was carried out using electrospray ionization (ESI) and was performed on a Micromass LCT Premier spectrometer. High-resolution mass spectroscopy (HRMS) was carried out using Bruker MicroTOF and Micromass GCT spectrometers under ESI or field ionization (FI) conditions, respectively.

Melting points were determined using a Reichert melting point apparatus and are uncorrected.

Experimental procedures

Chavibetol (Compound 6)

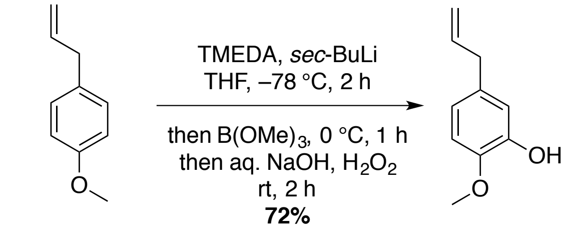

4-Allylanisole (8.00 g, 54.0 mmol) and TMEDA (8.0 mL, 54.0 mmol) were dissolved in THF (180 mL) and cooled to −78 °C. *s*-BuLi (1.4 M in cyclohexane, 50.1 mL, 70.2 mmol) was added dropwise over 15 min and the resulting mixture was stirred at −78 °C for 2 h. Then the cooling bath was removed. After 15 min the reaction mixture was put into an ice bath and stirred for additional 10 min before $B(OMe)_3$ (9.0 mL, 81.0 mmol) was added in one portion and the mixture was stirred for a further 1 h at 0 °C. NaOH (1.0 M in water, 54.0 mL, 54.0 mmol) and H_2O_2 (30 wt. % in water, 26.0 mL, 255 mmol) were added and the mixture was warmed to room temperature and stirred for 2 h. The reaction was quenched by addition of sat. aq. Na_2SO_3 (100 mL) and poured into sat. aq. NH_4Cl (100 mL). The mixture was extracted with Et_2O (3 × 100 mL) and the combined organic layers were washed with brine (50 mL), dried over $MgSO_4$, filtered, and concentrated under reduced pressure. The residue was purified by silica gel chromatography (EtOAc/PE 1:20 → 1:10) to give chavibetol (6.37 g, 72%) as a colorless liquid.

From extraction: Ground leaves of *Piper betle* (dried, 200 g, from Jawa Island, Indonesia) was mixed with NaOH (0.5 M in water, 1.5 L) and the container was flushed with nitrogen gas. The mixture was left standing at room temperature for 2 days with gentle agitation every 12 h. Then the mixture was filtered and the filter cake was thoroughly washed with NaOH (0.5 M in water, 500 mL). Silica gel (100 g) was added to the filtrate and, with vigorous stirring, pH of the mixture was adjusted to 3 by adding concentrated aq. HCl. The aqueous suspension was filtered and the filter cake was washed with EtOH (500 mL). The filtrate was saturated with NaCl and extracted with Et_2O (3 × 300 mL). The combined organic layers were dried over Na_2SO_4 and concentrated under reduced pressure. The residue was purified by silica gel chromatography (Et_2O/PE 1:4) to give chavibetol (4.14 g, 2.1% dry weight of the leaves) as a pale yellow oil.

^{1}H NMR (500 MHz, $CDCl_3$) δ 6.79 (d, J = 8.2 Hz, 1H), 6.79 (d, J = 2.1 Hz, 1H), 6.67 (dd, J = 8.2, 2.1 Hz, 1H), 5.95 (ddt, J = 16.8, 10.0, 6.8 Hz, 1H), 5.59 (s, 1H), 5.09-5.03 (m, 2H), 3.87 (s, 3H), 3.30 (d, J = 6.8 Hz, 2H); ^{13}C NMR (125 MHz, $CDCl_3$) δ 145.7, 145.1, 137.8, 133.6, 120.0, 115.7, 115.0, 110.8, 56.2, 39.7; IR (film, cm^{-1}) ν_{max} 3516, 3077, 2938, 2840, 1509, 1270; MS (ESI) 187.1 [M+Na]$^+$. These data are consistent with that previously published for this compound.[iii]

Compound 9

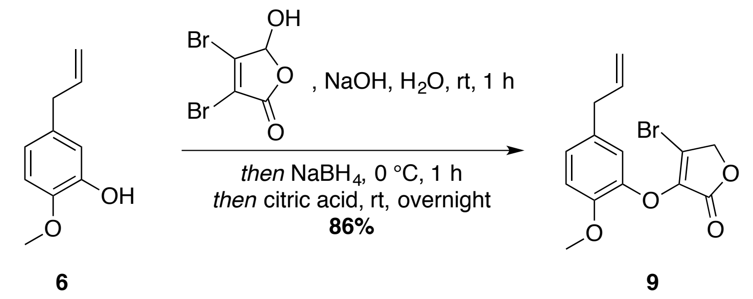

Mucobromic acid (10.5 g, 40.7 mmol) was suspended in water (12.5 mL) under sonication and vigorous agitation before NaOH (1.0 M in water, 42.5 mL, 42.5 mmol) was added dropwise. The resulting slightly hazy solution was added to a solution of chavibetol (1.33 g, 8.10 mmol) in NaOH (0.75 M in water, 12.5 mL, 9.38 mmol) in one portion to give a yellow emulsion. Aqueous NaOH (1.0 M) was added every 5 minutes for 1 h to keep the reaction mixture as a slightly hazy solution (approx. 17 mL required). Then the solution was cooled to 0 ºC before $NaBH_4$ (1.38 g, 36.5 mmol) was added in one portion and the solution was stirred for 1 h at 0 ºC. The reaction mixture was quenched by adding citric acid (3.0 M in water, 38 mL, 114 mmol) at 0 ºC. After the addition, $CHCl_3$ (75 mL) was added and the biphasic mixture was stirred overnight at room temperature. Then the organic layer was separated and the aqueous layer was further extracted with $CHCl_3$ (2 × 60 mL). The combined organic layers were dried over Na_2SO_4 and concentrated under reduced pressure. The residue was purified by silica gel chromatography (EtOAc/PE 1:5) to give butyrolactone **9** (2.25 g, 86%) as a colorless solid, and recovered chavibetol (0.130 g, 10%).

^{1}H NMR (500 MHz, $CDCl_3$) δ 6.96 (dd, J = 8.3, 2.1 Hz, 1H), 6.90 (d, J = 2.1 Hz, 1H), 6.87 (d, J = 8.3 Hz, 1H), 5.92 (ddt, J = 16.8, 10.2, 6.6 Hz, 1H), 5.08-5.02 (m, 2H), 4.81 (s, 2H), 3.82 (s, 3H), 3.31 (d, J = 6.6 Hz, 2H); ^{13}C NMR (125 MHz, $CDCl_3$) δ 165.1, 149.1, 142.9, 141.3, 137.3, 132.8, 126.0, 120.5, 116.2, 115.6, 112.7, 70.1, 56.4, 39.3; IR (film, cm^{-1}) ν_{max} 3027, 2933, 2874, 1775, 1670, 1509, 1261, 1165, 1085, 1024; HRMS (ESI) calcd for $C_{14}H_{13}O_4BrNa$ [M+Na]$^+$ 346.9889, found 346.9890; m.p. 34-35 ºC.

tert-Butyl methyl(vinyl)carbamate

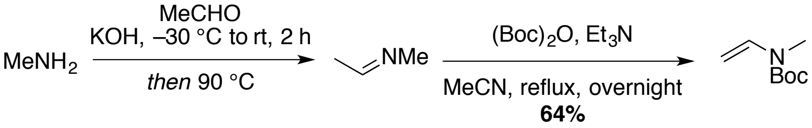

Acetaldehyde (33.8 mL, 0.6 mol) was added to methylamine (18.6 g, 0.6 mol) dropwise under vigorous stirring at −30 ºC. Then the viscous mixture was warmed to −10 ºC and stirred for 0.5 h before KOH (15.0 g) was added in one portion. The mixture was gradually warmed to room temperature over 1 h and the aqueous layer was separated. KOH (10.0 g) was added to the organic layer and the mixture was stirred at room temperature for 1 h. Then the organic layer (containing mainly *N*-ethylidenemethylamine trimer) was transferred to a distillation apparatus fitted with a Vigreux column and dry-ice trap. The temperature of heating bath was set to 90 ºC and the fraction with boiling range 28–34 ºC was collected to give *N*-ethylidenemethylamine (24.7 g, 0.433 mol) as a colorless liquid. This compound was used immediately without storage by adding to a mixture of $(Boc)_2O$ (79.1 g, 0.363 mol) and triethylamine (60.4 mL, 0.433 mol) in MeCN (180 mL) at 0 ºC. After the addition, the reaction mixture was warmed to room temperature and stirred for 1 h. Then the reaction mixture was kept at reflux overnight. Solvent was then removed under reduced pressure (100 mmHg, 40 ºC) until approx. 70 mL of residue left. The residue was purified by silica gel chromatography (Et_2O/PE (boiling range 30–40 ºC) 1:40) to give the title compound (36.5 g, 64% based on $(Boc)_2O$) as a colorless liquid.

^{1}H NMR (500 MHz, d_8-toluene, 363 K) δ 7.26 (dd, J = 15.8, 9.3 Hz, 1H), 4.07 (d, J = 9.3 Hz, 1H), 4.06 (d, J = 15.8 Hz, 1H), 2.79 (s, 3H), 1.38 (s, 9H); ^{13}C NMR (125 MHz, d_8-toluene, 363 K) δ 152.9, 135.0, 90.0, 80.5, 29.7, 28.2; IR (film, cm^{-1}) ν_{max} 2980, 2933, 1708, 1626, 1319, 1147; HRMS (FI) calcd for $C_8H_{15}NO_2$ $[M]^+$ 157.1103, found 157.1107.

Compound 5

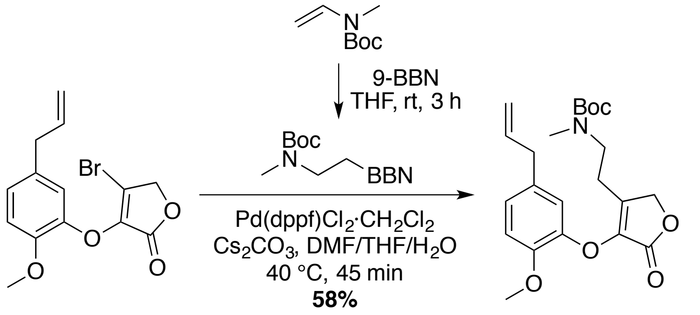

9 5

9-BBN (0.5 M in THF, 39.0 mL, 19.5 mmol) was added to *tert*-Butyl methyl(vinyl)carbamate (3.84 g, 24.4 mmol) at 0 ºC. After the addition, the solution was warmed to room temperature and stirred for 3 h before water (6.50 mL, degassed) was added. The content in the flask was transferred to a mixture of butyrolactone **9** (5.30 g, 16.3 mmol), Pd(dppf)Cl_2·CH_2Cl_2 (1.33 g, 1.63 mmol), and Cs_2CO_3 (10.6 g, 32.6 mmol) in DMF (110 mL, degassed) and the flask was further washed with DMF (20 mL, degassed). The brown biphasic mixture was warmed to 40 ºC and stirred for 45 min. Then the reaction mixture was diluted with water (250 mL), brine (250 mL), and extracted with Et_2O (3 × 300 mL). The combined organic layers were washed with brine (100 mL), dried over Na_2SO_4 and concentrated under reduced pressure. The residue was purified by silica gel chromatography (EtOAc/PE 1:2) to give butenolide **5** (3.81 g, 58%) as an off-white solid.

^{1}H NMR (500 MHz, d_8-toluene, 363 K) δ 6.94 (d, *J* = 2.1 Hz, 1H), 6.73 (dd, *J* = 8.3, 2.1 Hz, 1H), 6.62 (d, *J* = 8.3 Hz, 1H), 5.86 (ddt, *J* = 16.9, 10.2, 6.6 Hz, 1H), 5.01-4.95 (m, 2H), 4.25 (s, 2H), 3.50 (s, 3H), 3.16 (d, *J* = 6.6 Hz, 2H), 3.10 (t, *J* = 7.0 Hz, 2H), 2.57 (s, 3H), 2.24 (t, *J* = 7.0 Hz, 2H), 1.39 (s, 9H); ^{13}C NMR (125 MHz, d_8-toluene, 363 K) δ 166.5, 155.3, 149.6, 145.9, 140.4, 138.7, 137.6, 133.5, 124.7, 119.9, 115.6, 114.3, 79.4, 68.1, 56.4, 46.5, 39.5, 33.8, 28.4, 24.0; IR (film, cm^{-1}) ν_{max} 2976, 2939, 2842, 1766, 1691, 1516, 1399, 1248, 1127; HRMS (ESI) calcd for $C_{22}H_{29}NO_6Na$ $[M+Na]^+$ 426.1886, found 426.1887; m.p. 78-80 ºC.

Compound 4

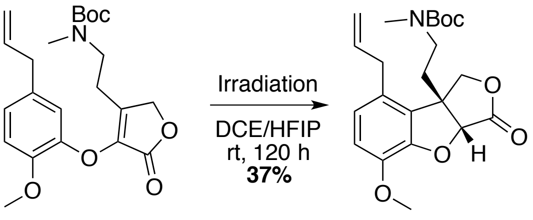

5 **4**

Butenolide **5** (2.80 g, 6.94 mmol) was dissolved in a mixture of dichloroethane and hexafluoroisopropanol (1:1 v/v, 350 mL, degassed) and irradiated with a 500 W high-pressure mercury-vapor lamp in a Pyrex immersion well reactor at room temperature for 120 h. Then the solvent was removed under reduced pressure and the residue was purified by silica gel chromatography (EtOAc/PE 1:2) to give benzofuran **4** (1.04 g, 37%) as a colorless amorphous solid, and recovered butenolide **5** (0.603 g, 22%).

^{1}H NMR (500 MHz, d_8-toluene, 363 K) δ 6.60 (d, *J* = 8.3 Hz, 1H), 6.57 (d, *J* = 8.3 Hz, 1H), 5.81 (ddt, *J* = 16.6, 10.0, 6.2 Hz, 1H), 4.99 (d, *J* = 10.0 Hz, 1H), 4.93 (d, *J* = 16.6 Hz, 1H), 4.65 (s, 1H), 4.30 (d, *J* = 9.2 Hz, 1H), 3.77 (d, *J* = 9.2 Hz, 1H), 3.56 (s, 3H), 3.13-3.03 (m, 2H), 2.90 (ddd, *J* = 13.9, 10.6, 4.9 Hz, 1H), 2.63 (ddd, *J* = 13.9, 10.4, 5.5 Hz, 1H), 2.49 (s, 3H), 1.92 (ddd, *J* = 13.6, 10.6, 5.5 Hz, 1H), 1.59 (ddd, *J* = 13.6, 10.4, 4.9 Hz, 1H), 1.40 (s, 9H); ^{13}C NMR (125 MHz, d_8-toluene, 363 K) δ 172.1, 155.2, 148.6, 144.5, 137.0, 128.6, 127.4, 124.3, 116.4, 116.2, 83.9, 79.3, 74.6, 56.8, 52.9, 45.5, 35.4, 34.4, 33.9, 28.5; IR (film, cm^{-1}) ν_{max} 2956, 2924, 2853, 1795, 1780, 1693, 1508, 1280, 1169, 1156, 1031; HRMS (ESI) calcd for $C_{22}H_{29}NO_6Na$ $[M+Na]^+$ 426.1886, found 426.1887.

Compound 11

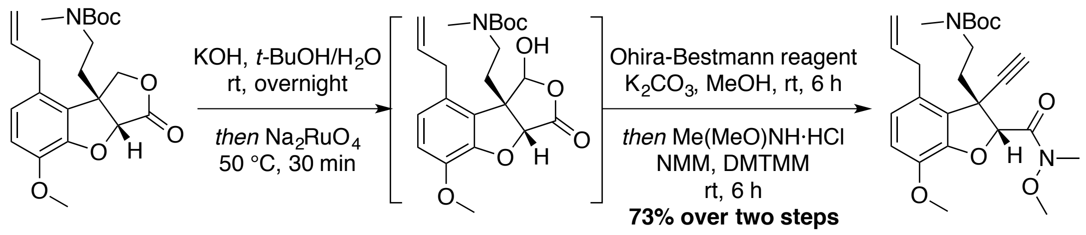

4 **10** **11**

KOH (1.0 M in water, 9.30 mL, 9.30 mmol) was added to a solution of benzofuran **4** (190 mg, 0.471 mmol) in *t*-BuOH (9.3 mL) and the biphasic mixture was stirred at room temperature overnight. Then Na_2RuO_4[iv] (0.0085 M in 1.0 M aq. NaOH, 61.0 mL, 0.519 mmol) was added in one portion and the reaction mixture was warmed to 50 ºC for 30 min. The black suspension was filtered and the filter cake was successively washed with water (10 mL) and IPA (20 mL). The filtrate was diluted with brine (50 mL), acidified with HCl (3.0 M in water, 30.0 mL, 90.0 mmol), and extracted with DCM (3 × 50 mL). The combined organic layers were dried over Na_2SO_4 and concentrated under reduced pressure. The resulting crude hemiacetal **10** was dissolved in MeOH (18.6 mL) followed by addition of Ohira-Bestmann reagent (181 mg, 0.942 mmol) and K_2CO_3 (325 mg, 2.35 mmol) at room temperature. The mixture was stirred for 6 h, then *N*,*O*-dimethylhydroxylamine hydrochloride (276 mg, 2.83 mmol), 4-methylmorpholine (0.31 mL, 2.83 mmol), and 4-(4,6-Dimethoxy-1,3,5-triazin-2-yl)-4-methyl-morpholinium chloride (628 mg, with ≤17 wt. % water, 1.88 mmol) were successively added and stirred for 6 h at room temperature. The solvent was then removed under reduced pressure and the residue was suspended in a mixture of HCl (1.0 M in water, 15.0 mL, 15.0 mmol) and brine (30 mL), and extracted with Et_2O (3 × 30 mL). The combined organic layers were dried over Na_2SO_4 and concentrated under reduced pressure. The residue was purified by silica gel chromatography (EtOAc/PE 1:1) to give amide **11** (158 mg, 73% from benzofuran **4**) as a colorless amorphous solid.

^{1}H NMR (500 MHz, d_8-toluene, 363 K) δ 6.68 (d, *J* = 8.3 Hz, 1H), 6.63 (d, *J* = 8.3 Hz, 1H), 6.00 (ddt, *J* = 16.9, 10.1, 6.5 Hz, 1H), 5.58 (s, 1H), 5.08 (ddt, *J* = 16.9, 1.7, 1.7 Hz, 1H), 5.03 (ddt, *J* = 10.1, 1.7, 1.4 Hz, 1H), 3.68 (s, 3H), 3.66-3.63 (m, 2H), 3.46 (ddd, *J* = 13.8, 11.3, 4.8 Hz, 1H), 3.37 (s, 3H), 3.16 (ddd, *J* = 13.8, 11.3, 4.8 Hz, 1H), 2.95 (s, 3H), 2.62 (s, 3H), 2.32 (ddd, *J* = 13.8, 11.3, 4.8 Hz, 1H), 2.17 (ddd, *J* = 13.8, 11.3, 4.8 Hz, 1H), 2.04 (s, 1H), 1.42 (s, 9H); ^{13}C NMR (125 MHz, d_8-toluene, 363 K) δ 169.9, 155.3, 149.7, 143.9, 137.9, 129.4, 127.9, 123.1, 116.3, 115.6, 86.8, 83.6, 79.0, 73.7, 60.7, 57.2, 48.6, 45.6, 39.0, 34.8, 34.4, 32.7, 28.5; IR (film, cm^{-1}) ν_{max} 3259, 2972, 2931, 1688, 1507, 1437, 1392, 1283, 1155; HRMS (ESI) calcd for $C_{25}H_{34}N_2O_6Na$ $[M+Na]^+$ 481.2309, found 481.2306.

Compound 3

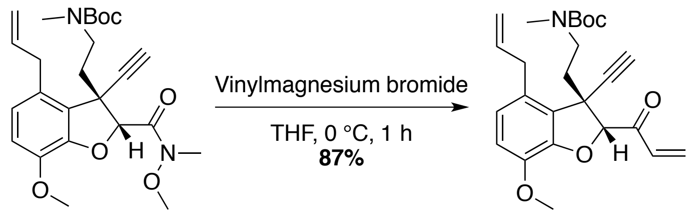

11 **3**

Vinylmagnesium bromide (1.0 M in THF, 1.40 mL, 1.40 mmol) was added dropwise to a solution of amide **11** (160 mg, 0.349 mmol) in THF (9.5 mL) at 0 ºC. The reaction mixture was stirred for 1 h at 0 ºC before quenched by addition of HCl (1.0 M in water, 30 mL, pre-cooled at 0 ºC). The mixture was further diluted with brine (20 mL) and extracted with Et_2O (4 × 20 mL). The combined organic layers were washed with brine (20 mL), dried over Na_2SO_4 and concentrated under reduced pressure. The residue was purified by silica gel chromatography (EtOAc/PE 1:3.5) to give vinyl ketone **3** (129 mg, 87%) as a colorless amorphous solid.

^{1}H NMR (500 MHz, d_8-toluene, 363 K) δ 6.68 (d, *J* = 8.4 Hz, 1H), 6.66 (d, *J* = 8.4 Hz, 1H), 6.63 (dd, *J* = 17.3, 10.6 Hz, 1H), 6.27 (dd, *J* = 17.3, 1.9 Hz, 1H), 5.98 (ddt, *J* = 16.9, 10.1, 6.5 Hz, 1H), 5.27 (dd, *J* = 10.6, 1.9 Hz, 1H), 5.08 (ddt, *J* = 16.9, 1.7, 1.6 Hz, 1H), 5.08 (s, 1H), 5.03 (ddt, *J* = 10.1, 1.7, 1.6 Hz, 1H), 3.62-3.58 (m, 2H), 3.59 (s, 3H), 3.38 (ddd, *J* = 13.9, 11.0, 5.0 Hz, 1H), 3.24 (ddd, *J* = 13.9, 11.0, 4.7 Hz, 1H), 2.64 (s, 3H), 2.34 (ddd, *J* = 13.5, 11.0, 5.0 Hz, 1H), 2.16-2.09 (m, 1H), 2.07 (s, 1H), 1.43 (s, 9H); ^{13}C NMR (125 MHz, d_8-toluene, 363 K) δ 194.5, 155.2, 148.4, 144.1, 137.4, 132.4, 129.8, 128.8, 128.1, 123.8, 116.0, 115.5, 92.7, 82.4, 79.1, 76.6, 56.6, 48.3, 45.5, 39.2, 34.8, 34.4, 28.5; IR (film, cm^{-1}) ν_{max} 3275, 3078, 2953, 2927, 2855, 1737, 1699, 1507, 1366, 1282, 1155; HRMS (ESI) calcd for $C_{25}H_{31}NO_5Na$ $[M+Na]^+$ 448.2094, found 448.2089.

Codeine (Compound 15)

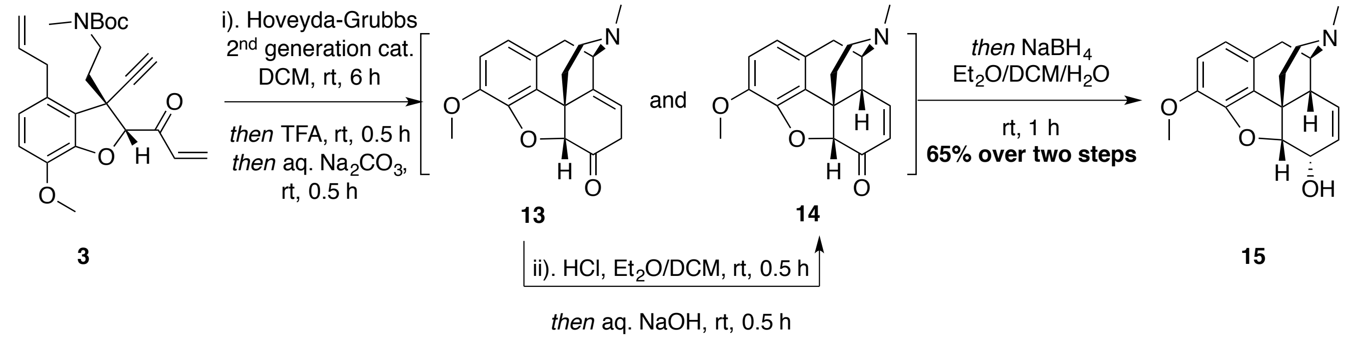

A solution of ketone **3** (40.5 mg, 0.095 mmol) and Hoveyda-Grubbs 2nd generation catalyst (3.0 mg, 0.005 mmol) in DCM (3.2 mL) was stirred for 6 h at room temperature. Then trifluoroacetic acid (0.29 mL, 3.79 mmol) was added and the reaction mixture was stirred for 30 min at room temperature. The reaction mixture was cooled to 0 ºC and sat. aq. Na_2CO_3 (1.6 mL) was added dropwise. After the addition, the biphasic mixture was warmed to room temperature and vigorously stirred for 30 min. Then the reaction mixture was diluted with water (2.5 mL) and the organic layer was separated. The aqueous layer was further extracted with DCM (4 × 2 mL). The combine organic layers were dried over Na_2SO_4 and concentrated under reduced pressure. The residue was purified by silica gel chromatography (MeOH/DCM 1:50 → 1:10) to give a mixture of neopinone **13** and codeinone **14** (20.4 mg, 0.069 mmol) as a yellow amorphous solid.

The above solid was dissolved in DCM (0.3 mL) followed by addition of HCl (2.0 M in Et_2O, 0.14 mL, 0.28 mmol) and stirred at room temperature for 30 min. Then the reaction mixture was cooled to 0 ºC before NaOH (0.4 M in water, 1.4 mL, 0.56 mmol) was added. The biphasic mixture was warmed to room temperature and vigorously stirred for 30 min. Then $NaBH_4$ (26.0 mg, 0.687 mmol) was added and the reaction mixture was stirred for 1 h at room temperature. The resulting mixture was diluted with 10% EtOH in DCM (1.5 mL) and the organic layer was separated. The aqueous layer was further extracted with 10% EtOH in DCM (3 × 2 mL). The combined organic layers were washed with brine (2 mL), dried over Na_2SO_4, and concentrated under reduced pressure. The residue was purified by silica gel chromatography (EtOH/DCM 1:10) to give codeine (18.5 mg, 65%) as an off-white solid.

^{1}H NMR (500 MHz, $CDCl_3$) δ 6.66 (d, J = 8.2 Hz, 1H), 6.57 (d, J = 8.2 Hz, 1H), 5.71 (dddd, J = 9.9, 3.2, 1.9, 1.3 Hz, 1H), 5.30 (ddd, J = 9.9, 3.2, 2.6 Hz, 1H), 4.89 (dd, J = 6.5, 1.3 Hz, 1H), 4.18 (ddd, J = 6.5, 3.2, 2.6 Hz, 1H), 3.84 (s, 3H), 3.35 (dd, J = 6.2, 3.2 Hz, 1H), 3.05 (d, J = 18.6 Hz, 1H), 2.67 (ddd, J = 3.2, 3.2, 1.9 Hz, 1H), 2.59 (dd, J = 12.5, 5.3 Hz, 1H), 2.44 (s, 3H), 2.40 (td, J = 12.5, 3.6 Hz, 1H), 2.29 (dd, J = 18.6, 6.2 Hz, 1H), 2.06 (td, J = 12.5, 5.3 Hz, 1H), 1.88 (ddd, J = 12.5, 3.6, 1.7 Hz, 1H); ^{13}C NMR (125 MHz, $CDCl_3$) δ 146.4, 142.4, 133.6, 131.2, 128.4, 127.4, 119.7, 113.0, 91.5, 66.5, 59.0, 56.5, 46.6, 43.3, 43.1, 41.0, 36.0, 20.5; IR (film, cm^{-1}) ν_{max} 3531, 3022, 2929, 2789, 1501, 1453, 1385, 1250, 1115, 1027; HRMS (ESI) calcd for $C_{18}H_{22}NO_3$ $[M+H]^+$ 300.1593, found 300.1594; m.p. 152-153 ºC.

Morphine (Compound 1)

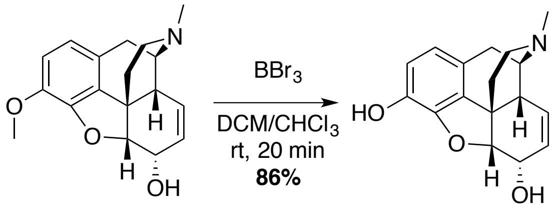

15 (±)-1

BBr_3 (1.0 M in DCM, 0.21 mL, 0.21 mmol) was added to a solution of codeine (10.5 mg, 0.035 mmol) in $CHCl_3$ (0.72 mL) at room temperature. After stirring for 20 min, the reaction was quenched by adding 10% aq. ammonium hydroxide (1.8 mL) at 0 ºC. The mixture was extracted with 10% EtOH in DCM (4 × 3 mL). The combined organic layers were dried over Na_2SO_4 and concentrated under reduced pressure. The residue was triturated with Et_2O (2 × 1 mL) under sonication to give morphine (8.6 mg, 86%) as an off-white solid.

^{1}H NMR (500 MHz, $CDCl_3$) δ 6.63 (d, J = 8.1 Hz, 1H), 6.50 (d, J = 8.1 Hz, 1H), 5.67 (dddd, J = 9.9, 3.1, 1.6, 1.1 Hz, 1H), 5.28 (ddd, J = 9.9, 3.3, 1.9 Hz, 1H), 4.89 (dd, J = 6.5, 1.1 Hz, 1H), 4.20 (ddd, J = 6.5, 3.1, 1.9 Hz, 1H), 3.41 (dd, J = 6.4, 3.2 Hz, 1H), 3.03 (d, J = 18.7 Hz, 1H), 2.73 (br, 1H), 2.66 (dd, J = 12.5, 4.7 Hz, 1H), 2.47 (s, 3H), 2.45 (td, J = 12.5, 3.4 Hz, 1H), 2.33 (dd, J = 18.7, 6.4 Hz, 1H), 2.12 (td, J = 12.5, 4.7 Hz, 1H), 1.88 (ddd, J = 12.5, 3.4, 1.7 Hz, 1H); ^{13}C NMR (125 MHz, $CDCl_3$) δ 145.2, 138.0, 133.2, 130.9, 128.5, 126.6, 120.2, 116.8, 91.9, 66.6, 59.1, 46.6, 43.3, 43.1, 40.7, 35.6, 20.7; IR (film, cm^{-1}) ν_{max} 3297, 2926, 1612, 1457, 1246, 1118, 1032; HRMS (ESI) calcd for $C_{17}H_{20}NO_3$ $[M+H]^+$ 286.1438, found 286.1440; m.p. 215-221 ºC (decomp.).

---

i A. B. Pangborn, M. A. Giardello, R. H. Grubbs, R. K. Rosen, F. J. Timmers, *Organometallics* **1996**, *15*, 1518.

ii W. L. F. Armarego, D. D. Perrin, *Purification of Laboratory Chemicals*, Butterworth-Heinemann, Oxford, 1996.

iii A. M. Eliansen, R. P. Thedford, K. R. Claussen, C. Yuan, D. Siegel, *Org. Lett.* **2014**, *16*, 3628.

iv R. M. Coates, P. D. Senter, W. R. Baker, *J. Org. Chem.* **1982**, *47*, 3597.

Table 1. Optimisation of the *B*-alkyl Suzuki-coupling.

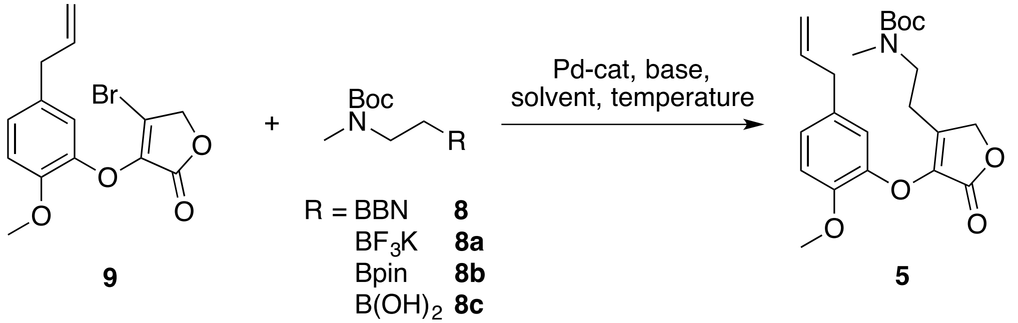

9 5

Entry[a]	*B*-Alkylreagent	Pd-cat.	Base	Solvent	H_2O	T (°C)	Yield[b]
1	**8** 1.5 eq.	$Pd(dppf)Cl_2{\cdot}CH_2Cl_2$ (10 mol%)	K_2CO_3 (2.0 eq.)	DMF/THF 2:1	28 eq.	rt	41%
2	**8** 1.5 eq.	$Pd(dppf)Cl_2{\cdot}CH_2Cl_2$ (10 mol%)	KF (4.0 eq.)	DMF/THF 2:1	5 eq.	70	-
3	**8** 1.5 eq.	$Pd(dppf)Cl_2{\cdot}CH_2Cl_2$ (10 mol%), $AsPh_3$ (20 mol%)	Cs_2CO_3 (2.0 eq.)	DMF/THF 2:1	28 eq.	rt	48%
4	**8** 1.5 eq.	$Pd(dppf)Cl_2{\cdot}CH_2Cl_2$ (10 mol%)	Cs_2CO_3 (2.0 eq.)	DMF/THF 2:1	28 eq.	rt	59%
5	**8** 1.5 eq.	$Pd(OAc)_2$ (10 mol%), RuPhos (20 mol%)	Cs_2CO_3 (2.0 eq.)	DMF/THF 2:1	28 eq.	rt	52%
6	**8** 1.5 eq.	XPhos Pd G3 (5 mol%)	Cs_2CO_3 (2.0 eq.)	DMF/THF 2:1	22 eq.	rt	25%
7	**8** 1.5 eq.	$Pd(OAc)_2$ (10 mol%), XPhos (20 mol%)	Cs_2CO_3 (2.0 eq.)	DMF/THF 2:1	22 eq.	rt	25%
8	**8** 1.1 eq.	$Pd(dppf)Cl_2{\cdot}CH_2Cl_2$ (10 mol%)	Cs_2CO_3 (2.0 eq.)	DMF/THF 2:1	13 eq.	rt	37%
9	**8** 1.5 eq.	$Pd(dppf)Cl_2{\cdot}CH_2Cl_2$ (10 mol%)	Cs_2CO_3 (2.0 eq.)	DMF/THF 2:1	22 eq.	rt	62%
10	**8** 1.5 eq.	$Pd(dppf)Cl_2{\cdot}CH_2Cl_2$ (10 mol%)	NaOH (2.0 eq.)	DMF/THF 2:1	22 eq.	rt	44%
11	**8a** 1.5 eq.	$Pd(dppf)Cl_2{\cdot}CH_2Cl_2$ (10 mol%)	Cs_2CO_3 (2.0 eq.)	DMF/THF 2:1	22 eq.	rt	28%
12	**8a** 1.5 eq.	$Pd(dppf)Cl_2{\cdot}CH_2Cl_2$ (10 mol%)	NaOH (2.0 eq.)	DMF/THF 2:1	22 eq.	rt	23%
13	**8b** 1.5 eq.	$Pd(dppf)Cl_2{\cdot}CH_2Cl_2$ (10 mol%)	Cs_2CO_3 (2.0 eq.)	DMF/THF 2:1	22 eq.	rt	31%
14	**8b** 1.5 eq.	$Pd(dppf)Cl_2{\cdot}CH_2Cl_2$ (10 mol%)	NaOH (2.0 eq.)	DMF/THF 2:1	22 eq.	rt	29%
15	**8c** 1.5 eq.	$Pd(dppf)Cl_2{\cdot}CH_2Cl_2$ (10 mol%)	Cs_2CO_3 (2.0 eq.)	DMF/THF 2:1	22 eq.	rt	-
16	**8c** 1.5 eq.	$Pd(dppf)Cl_2{\cdot}CH_2Cl_2$ (10 mol%)	NaOH (2.0 eq.)	DMF/THF 2:1	22 eq.	rt	
17	**8** 1.2 eq.	$Pd(dppf)Cl_2{\cdot}CH_2Cl_2$ (10 mol%)	Cs_2CO_3 (2.0 eq.)	DMF/THF 3.3:1	22 eq.	rt	61%
18	**8** 1.2 eq.	$Pd(dppf)Cl_2{\cdot}CH_2Cl_2$ (10 mol%)	Cs_2CO_3 (2.0 eq.)	DMF/THF 3.3:1	22 eq.	40	63%
19[c]	**8** 1.2 eq.	$Pd(dppf)Cl_2{\cdot}CH_2Cl_2$ (10 mol%)	Cs_2CO_3 (2.0 eq.)	DMF/THF 3.3:1	22 eq.	40	58%

All reactions were carried out under inert atmosphere following the procedure described in the experimental section. The reactions were monitored via TLC and stirred until the starting material was consumed. [a] Reactions were carried out on 20-200 mg scale, unless otherwise stated. [b] Isolated yield. [c] The reaction was carried out on 5.30 g scale.

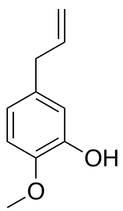

Chavibetol
^{1}H NMR, $CDCl_3$, 500 MHz

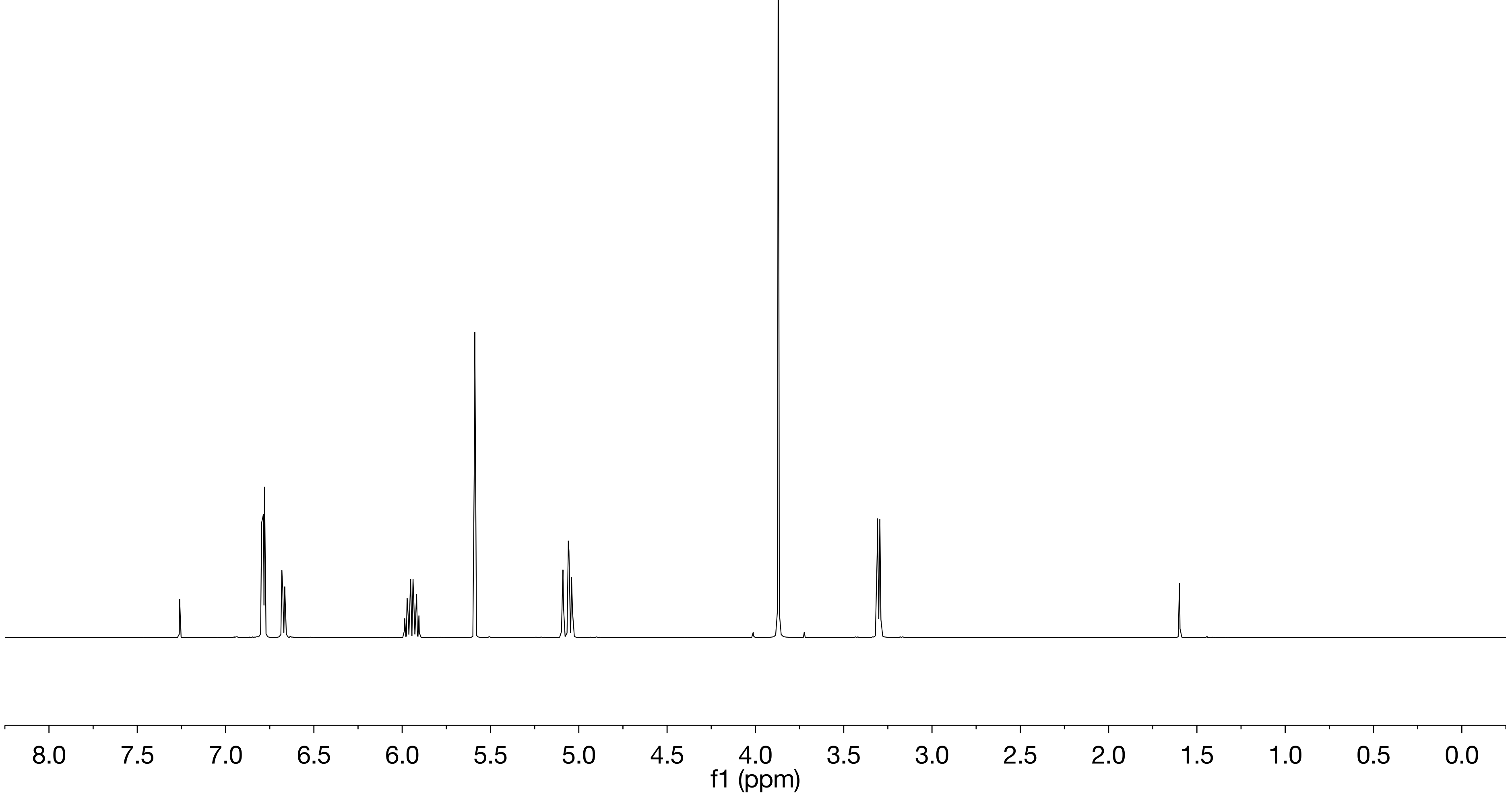

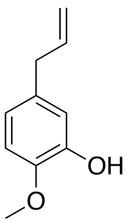

Chavibetol
^{13}C NMR, $CDCl_3$, 125 MHz

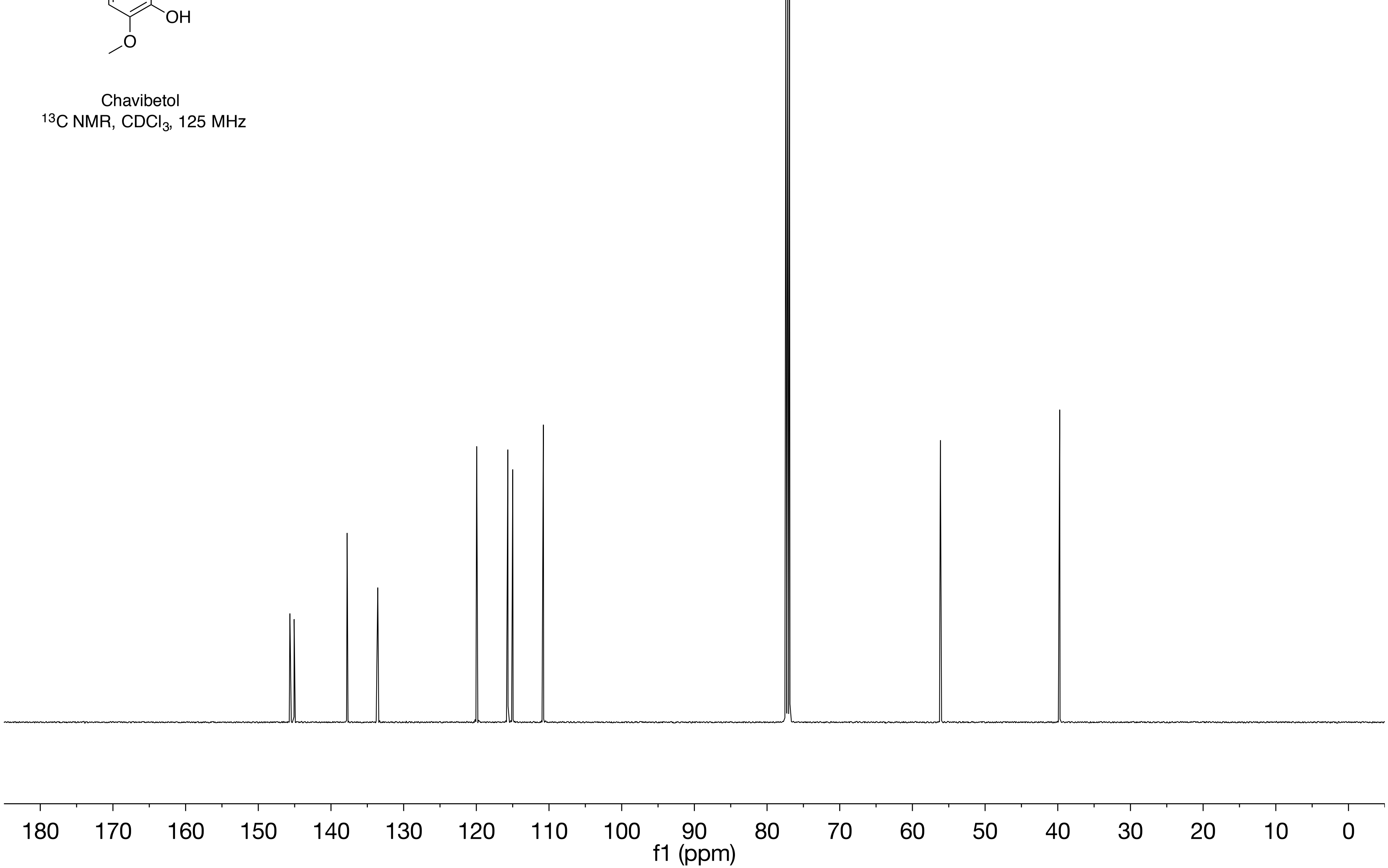

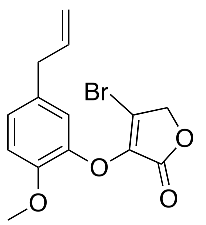

9
^{1}H NMR, $CDCl_3$, 500 MHz

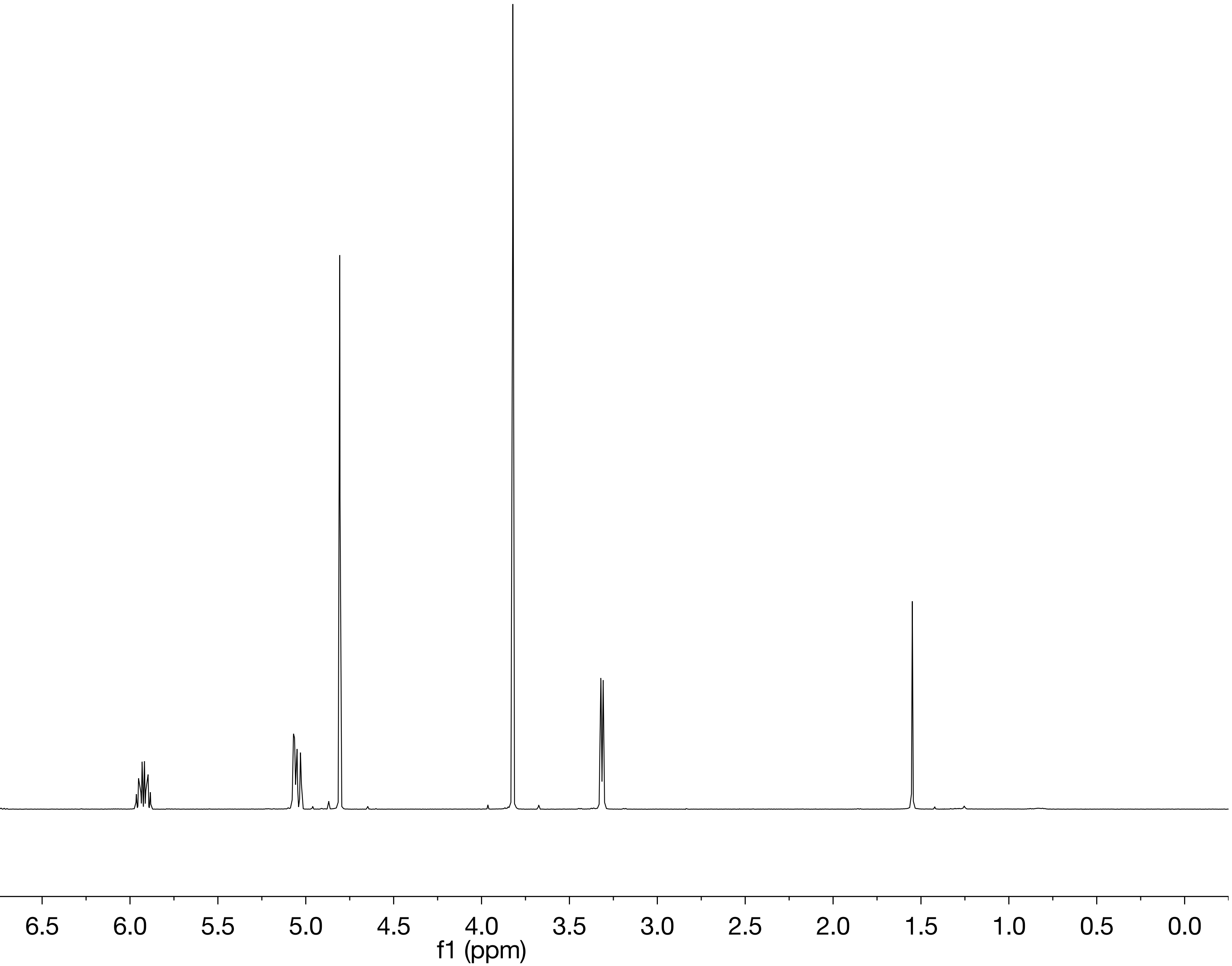

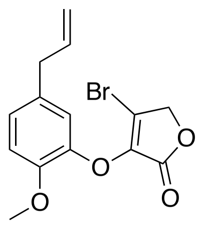

9

^{13}C NMR, $CDCl_3$, 125 MHz

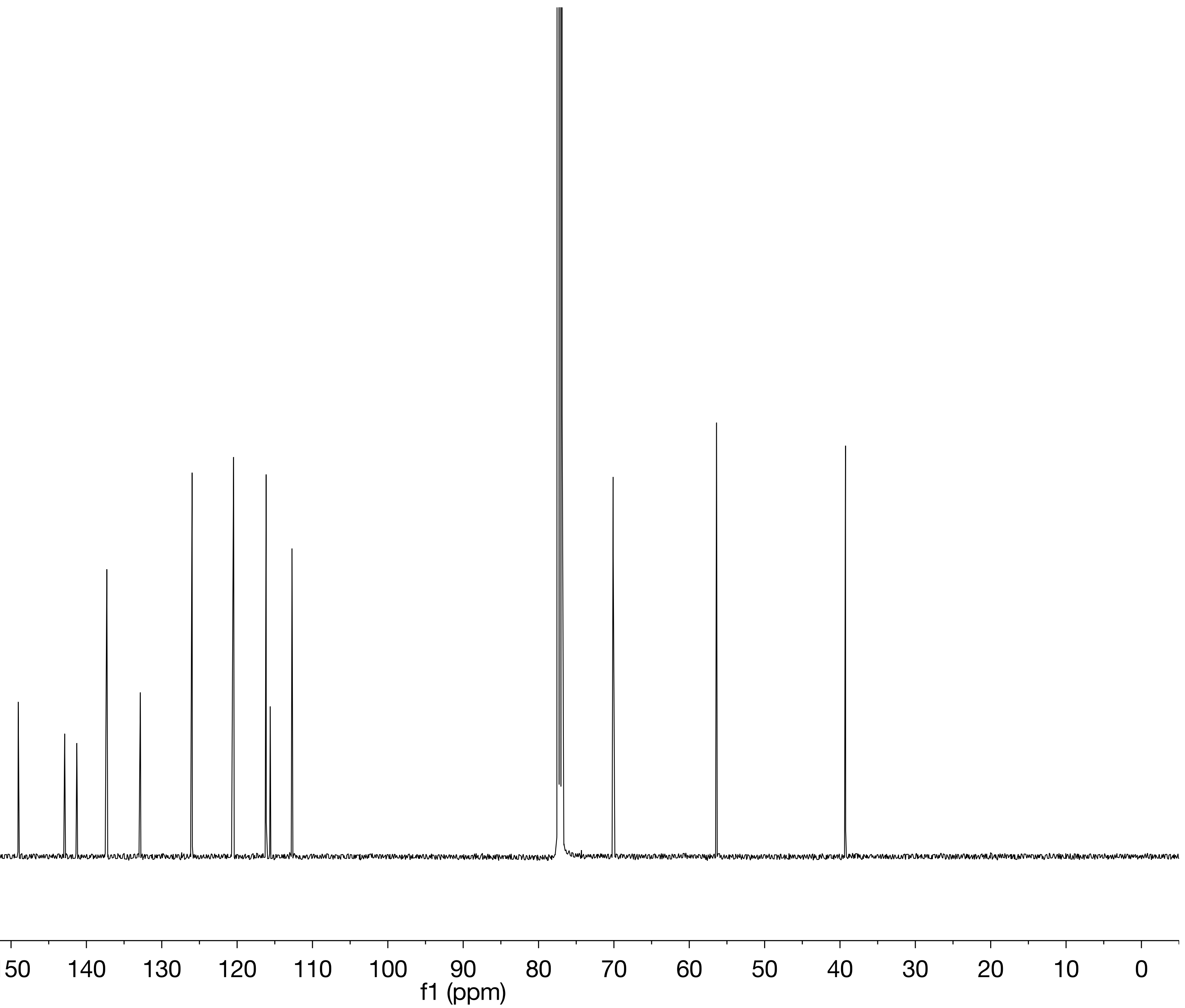

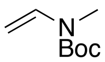

tert-Butyl methyl(vinyl)carbamate
^{1}H NMR, Toluene-d_8, 500 MHz, 363 K

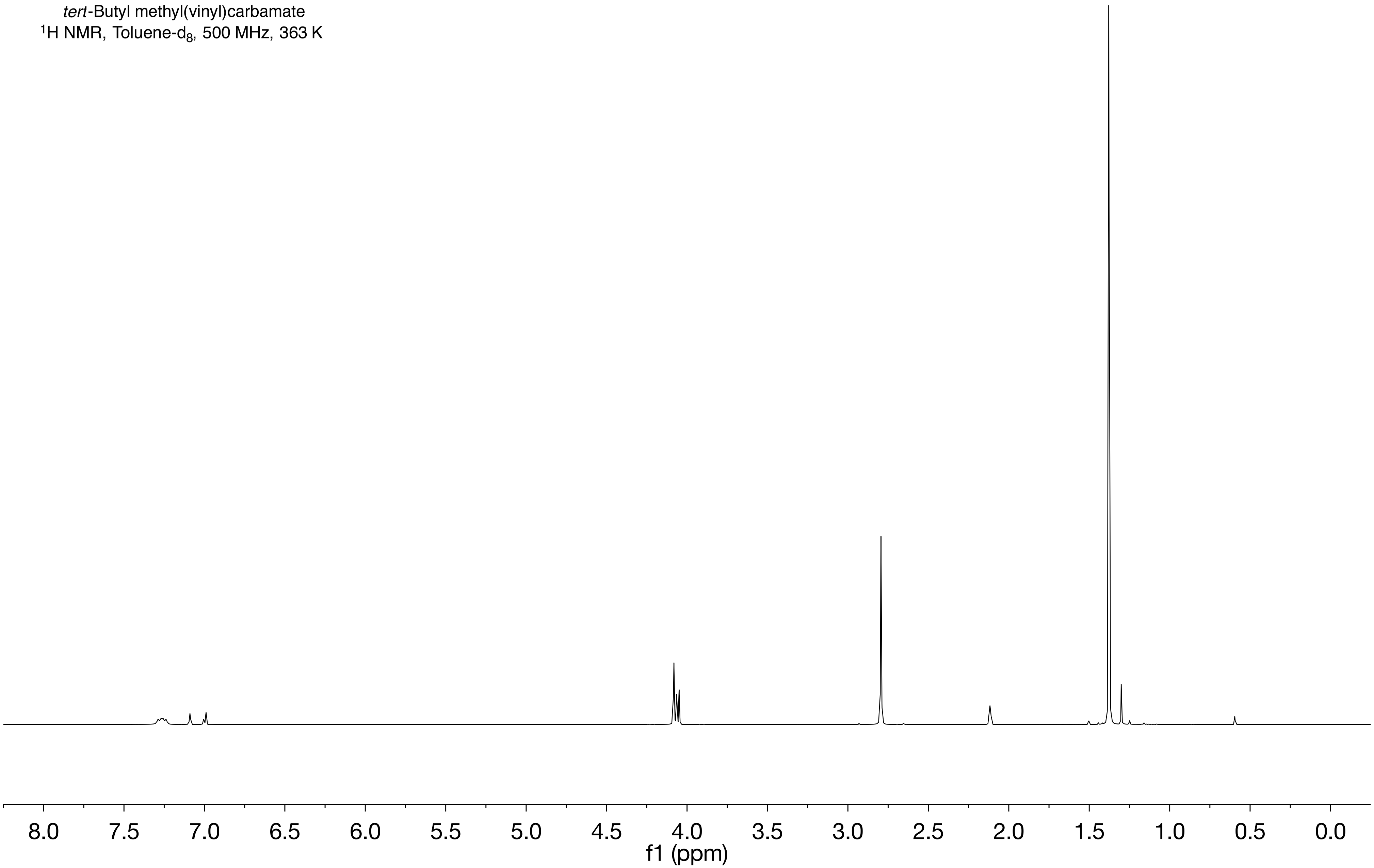

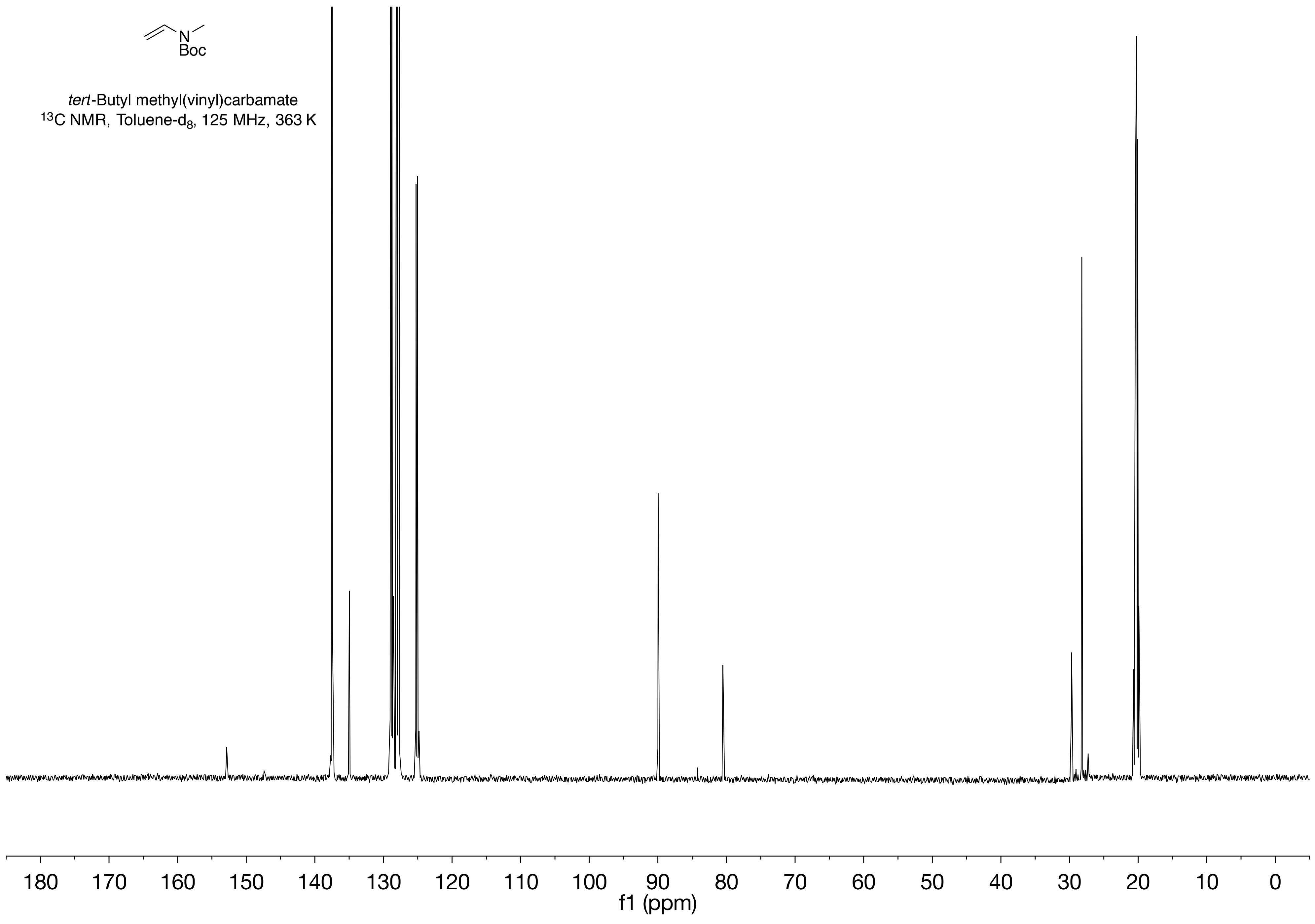
N
Boc
tert-Butyl methyl(vinyl)carbamate
^{13}C NMR, Toluene-d_8, 125 MHz, 363 K
180
170
160
150
140
130
120
110
100
90
80
70
60
50
40
30
20
10
0
f1 (ppm)

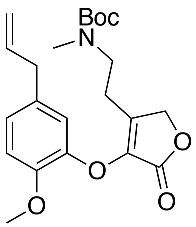

5

^{1}H NMR, Toluene-d_8, 500 MHz, 363 K

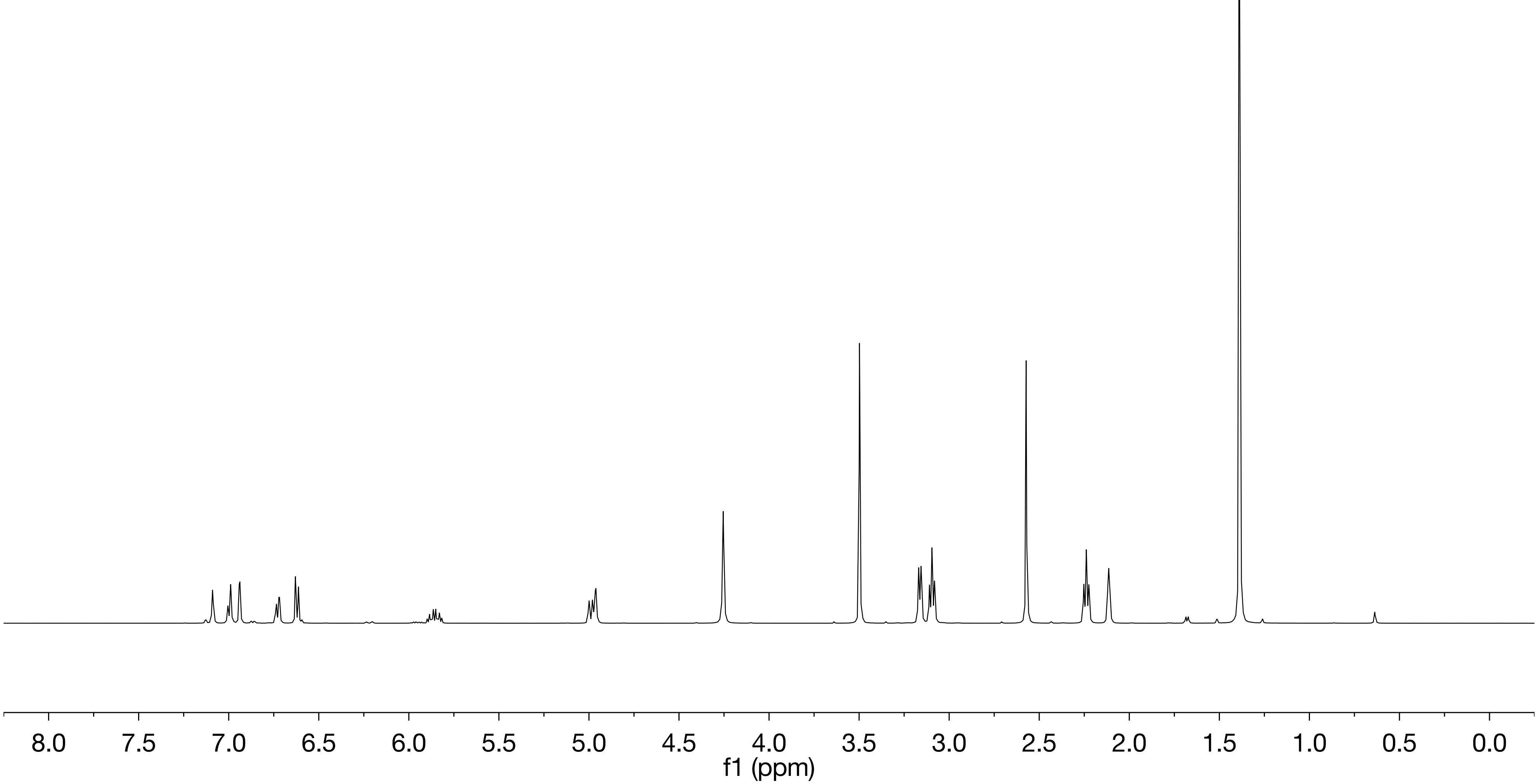

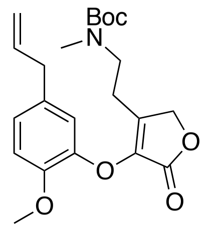

5

^{13}C NMR, Toluene-d_8, 125 MHz, 363 K

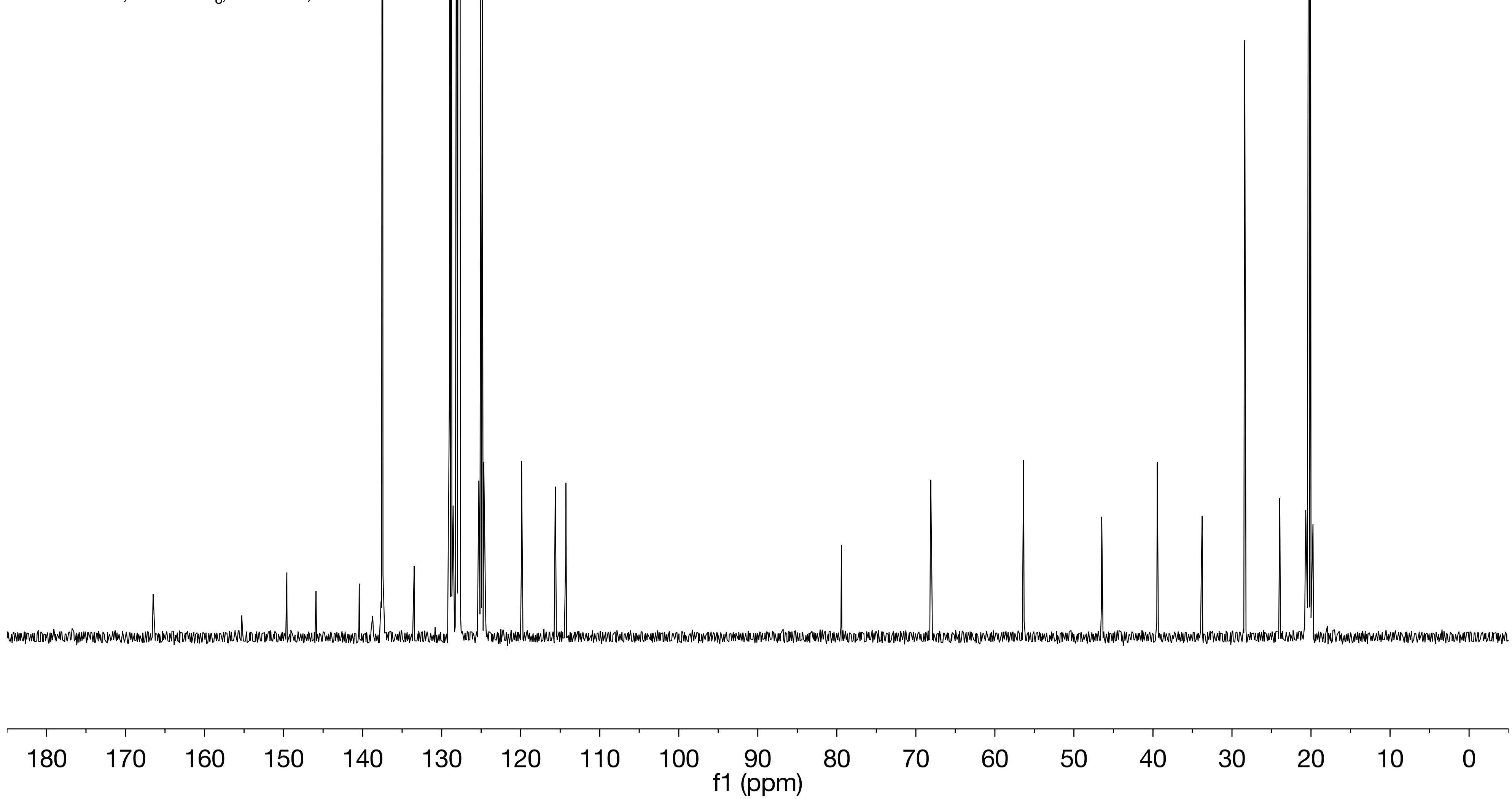

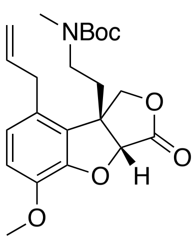

4
^{1}H NMR, Toluene-d_8, 500 MHz, 363 K

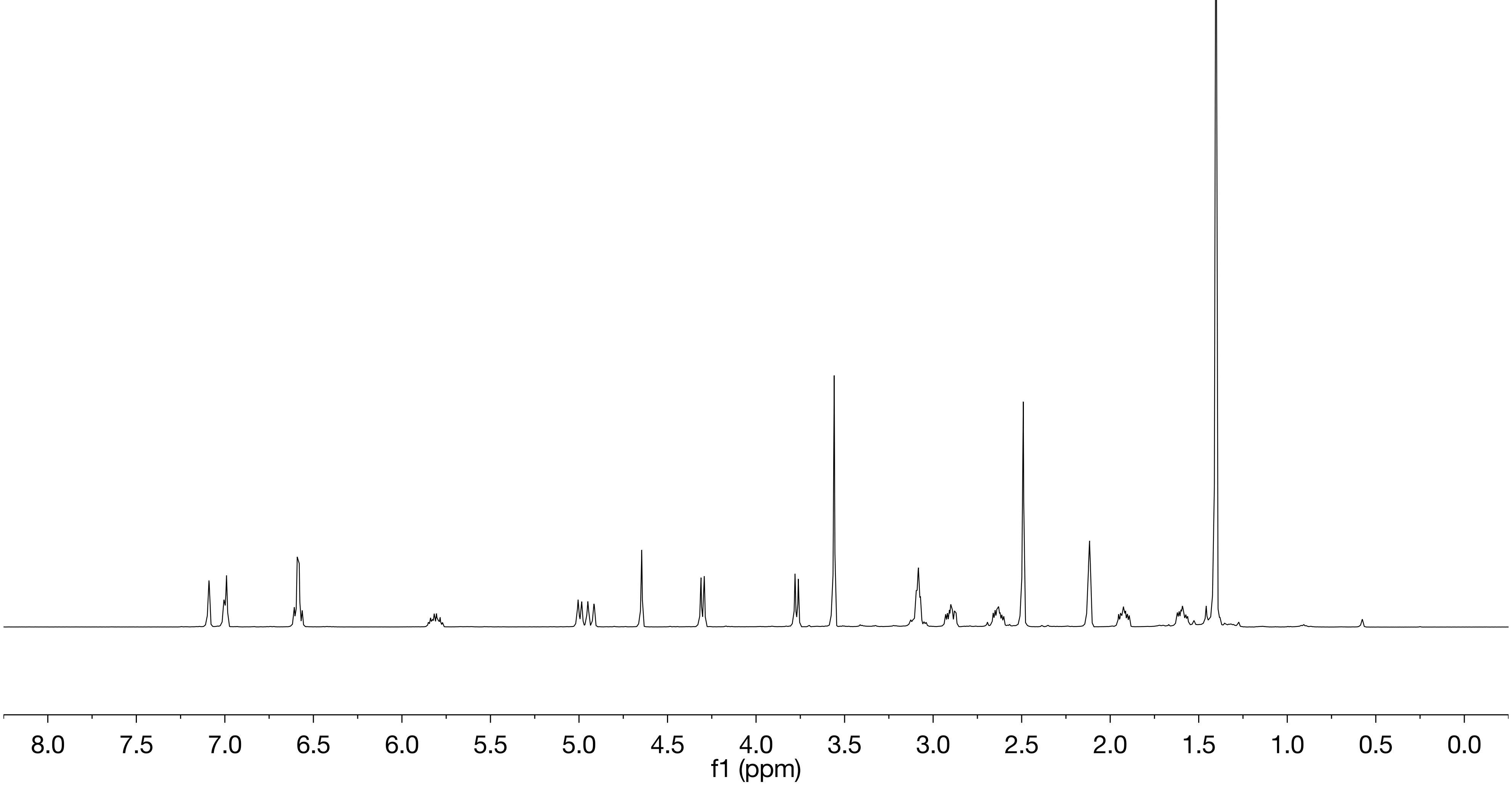

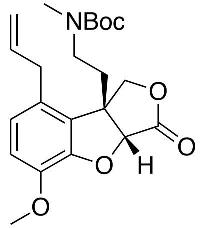

4

^{13}C NMR, Toluene-d_8, 125 MHz, 363 K

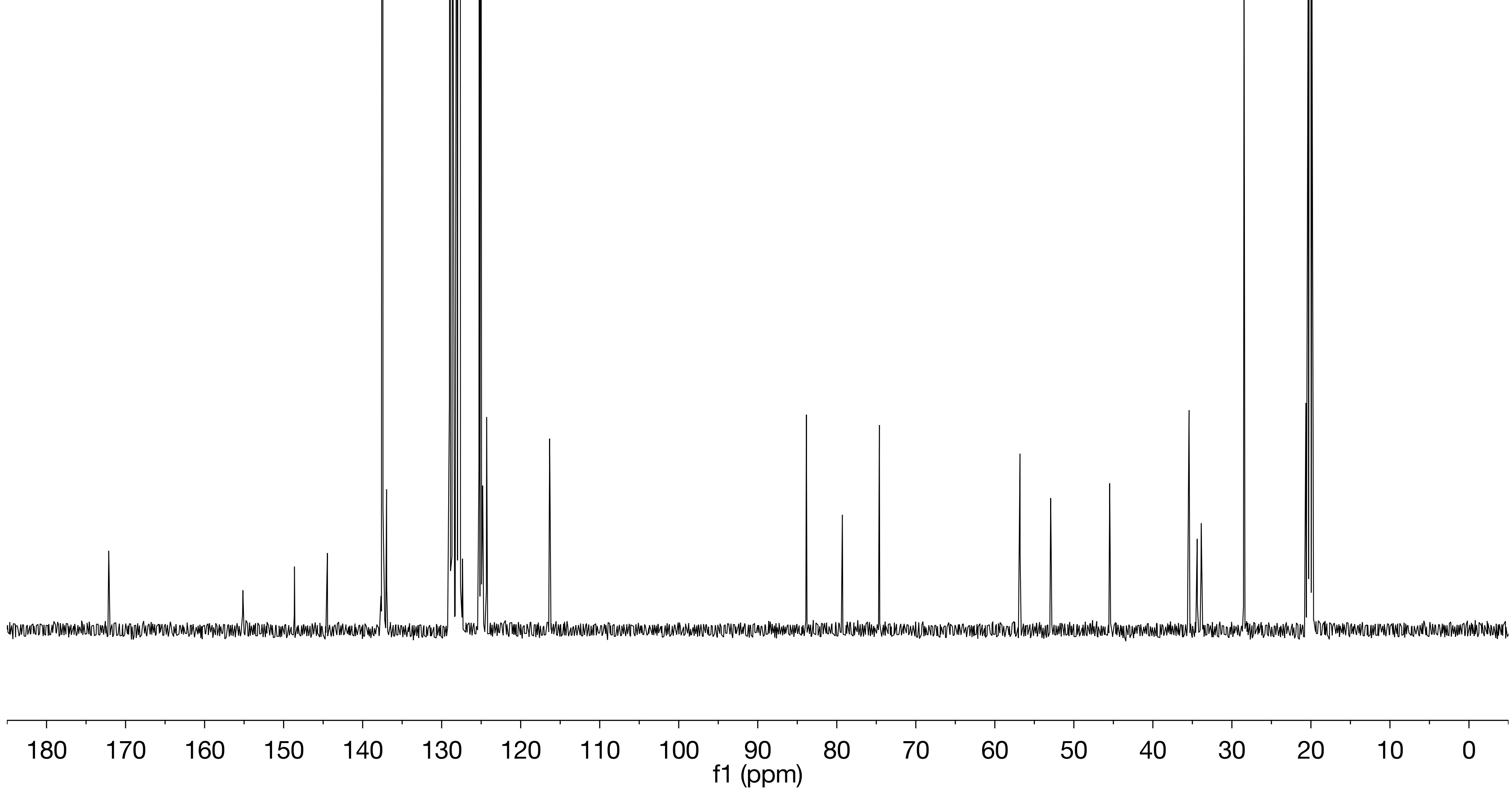

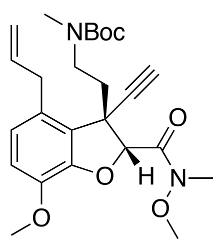

11
^{1}H NMR, Toluene-d_8, 500 MHz, 363 K

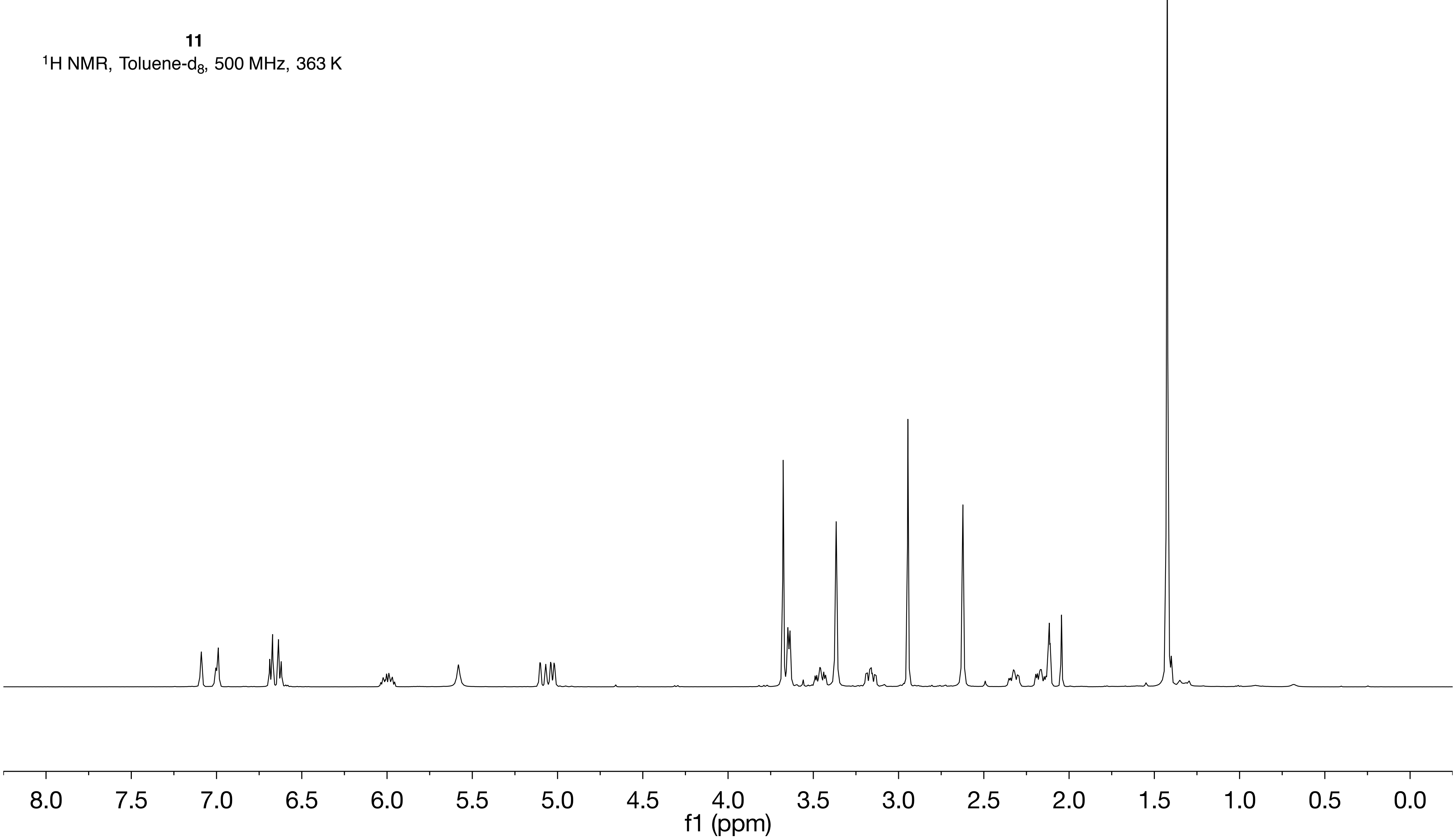

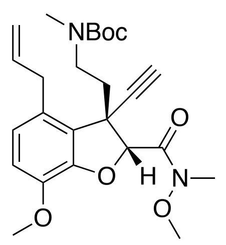

11

^{13}C NMR, Toluene-d_8, 125 MHz, 363 K

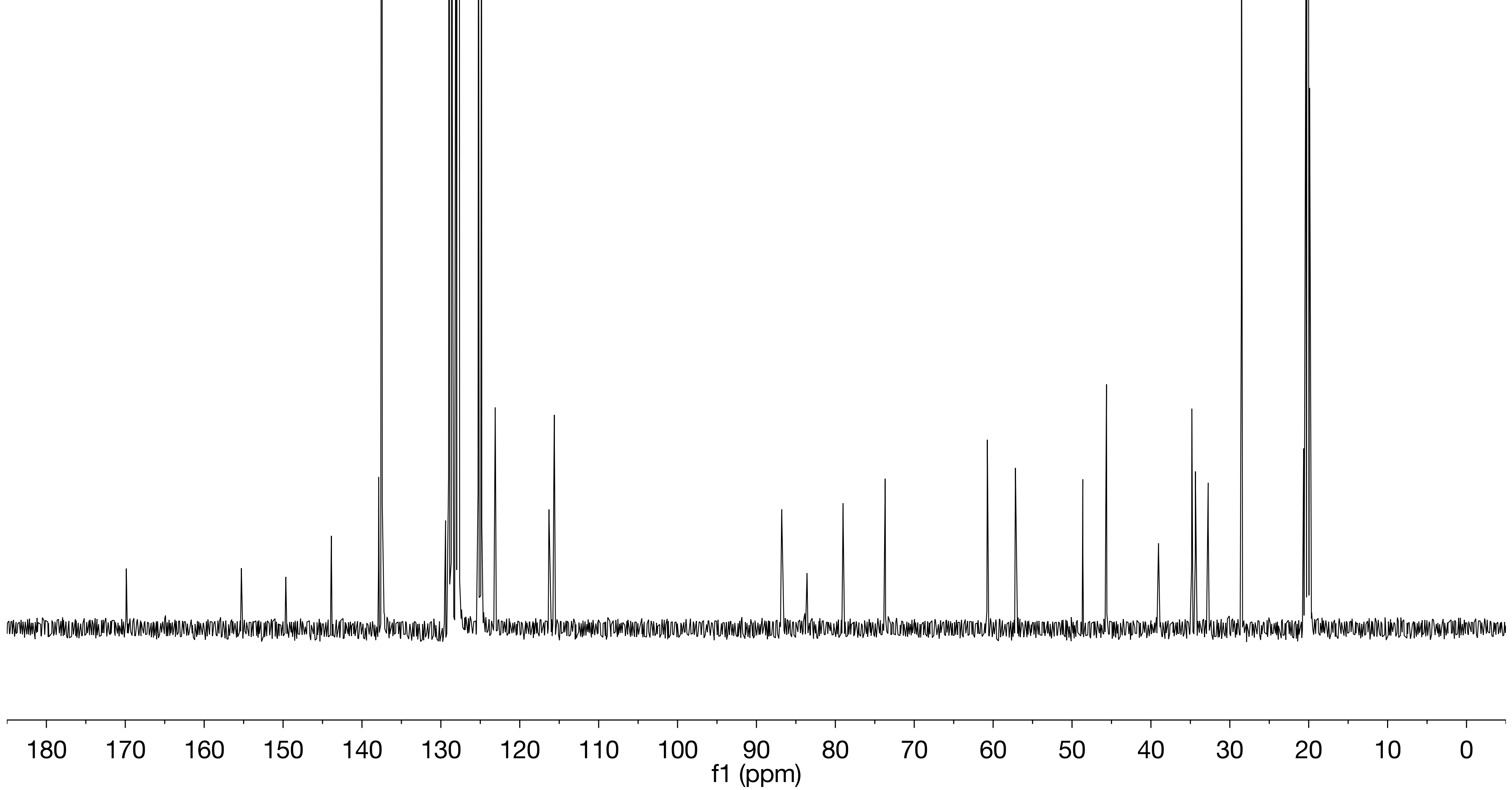

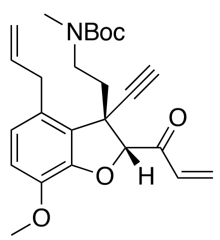

3

^{1}H NMR, Toluene-d_8, 500 MHz, 363 K

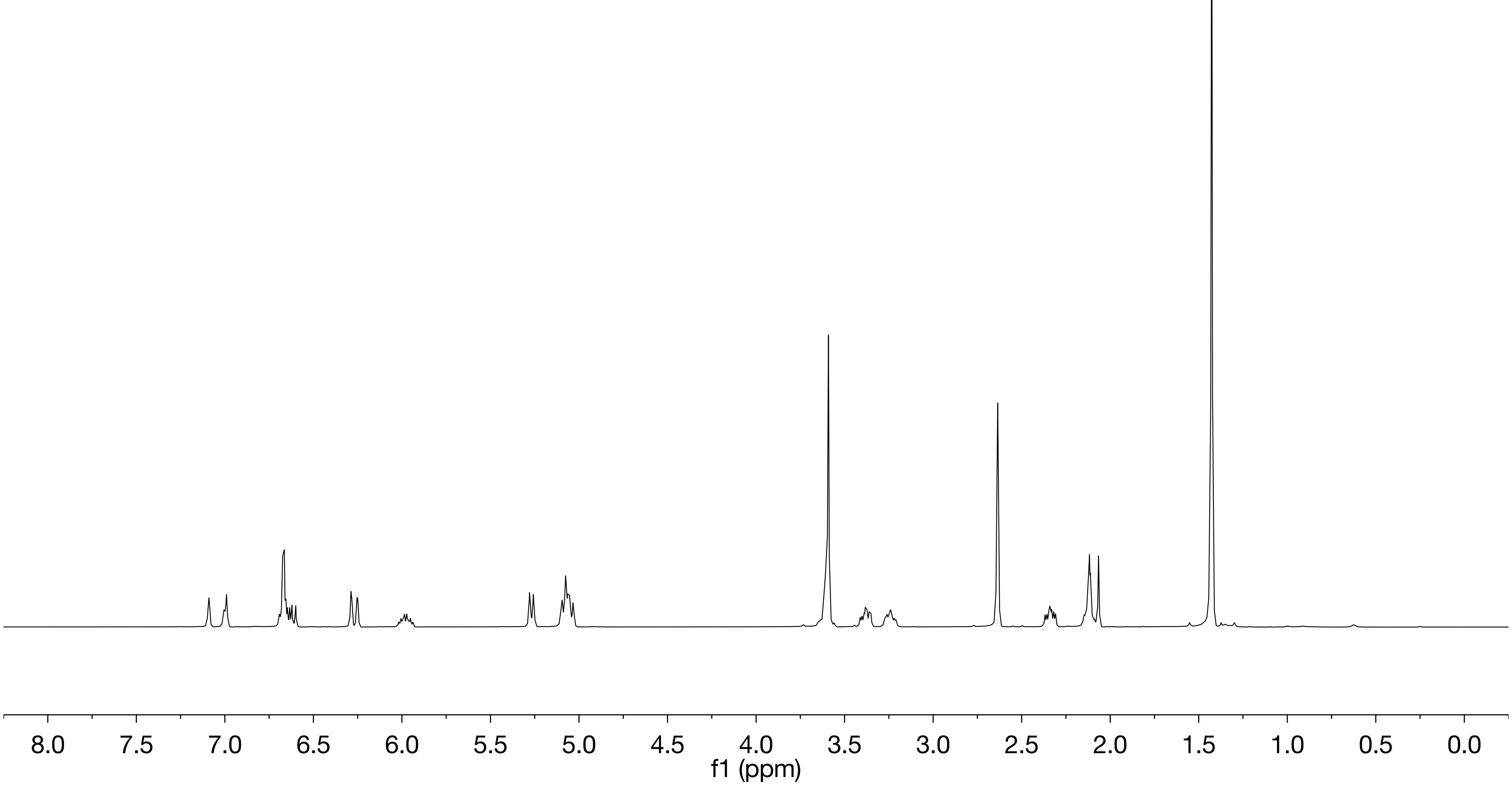

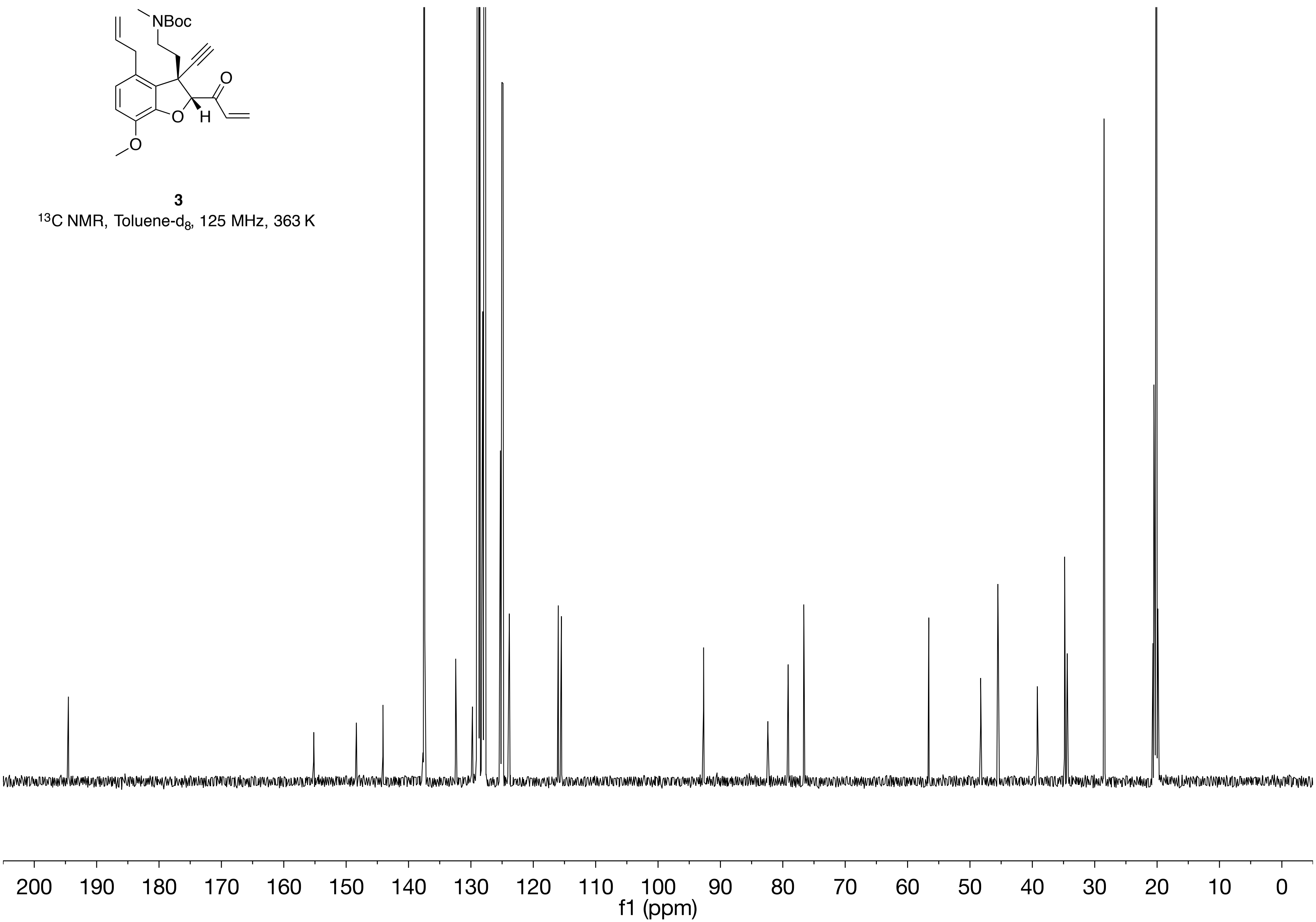

3

^{13}C NMR, Toluene-d_8, 125 MHz, 363 K

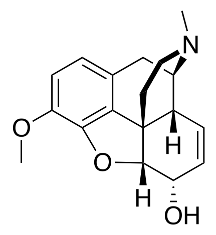

Codeine
^{1}H NMR, $CDCl_3$, 500 MHz

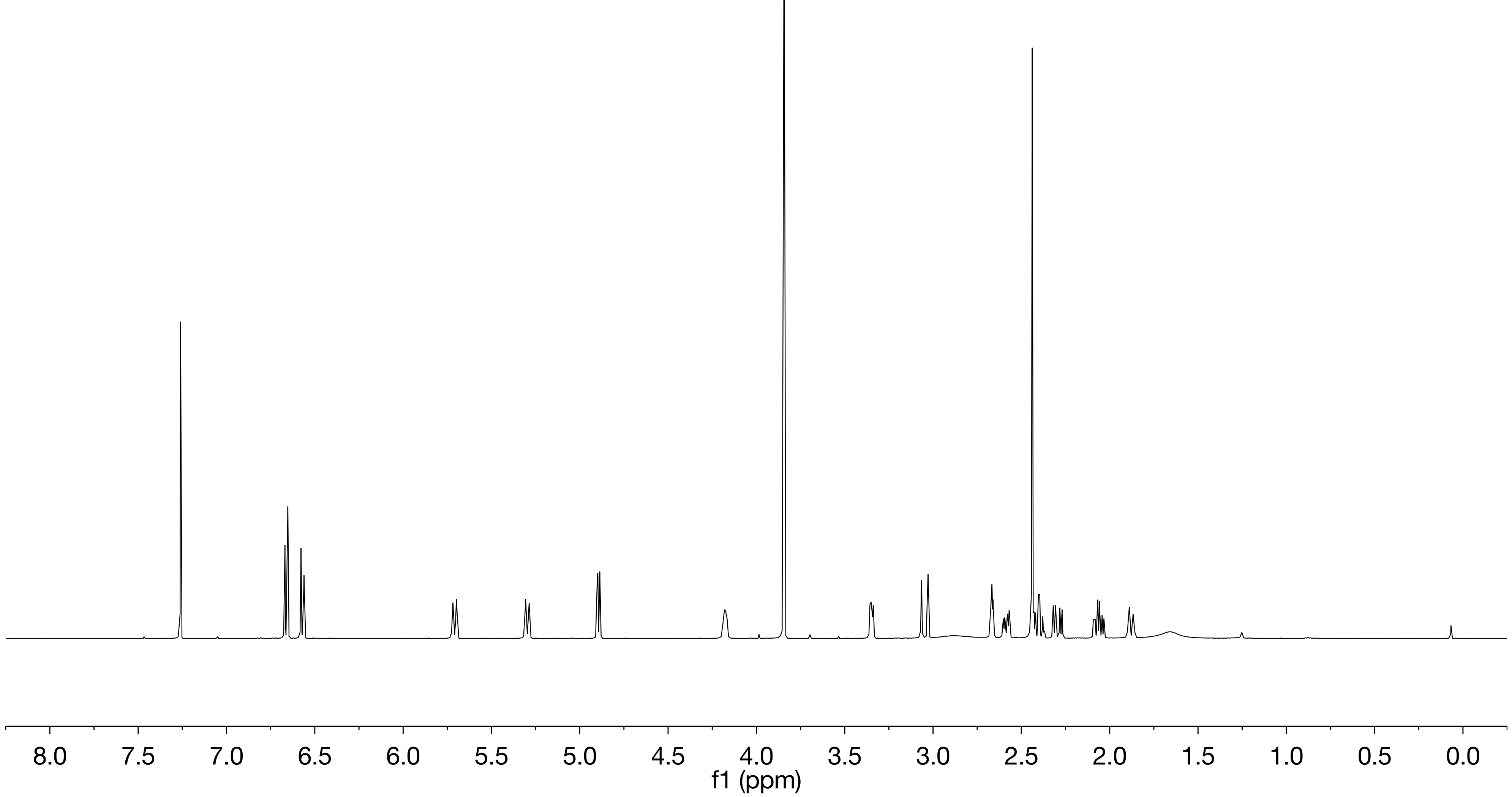

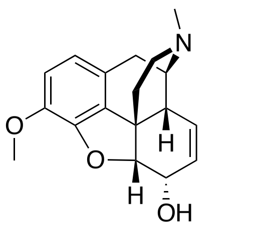

Codeine
^{13}C NMR, $CDCl_3$, 125 MHz

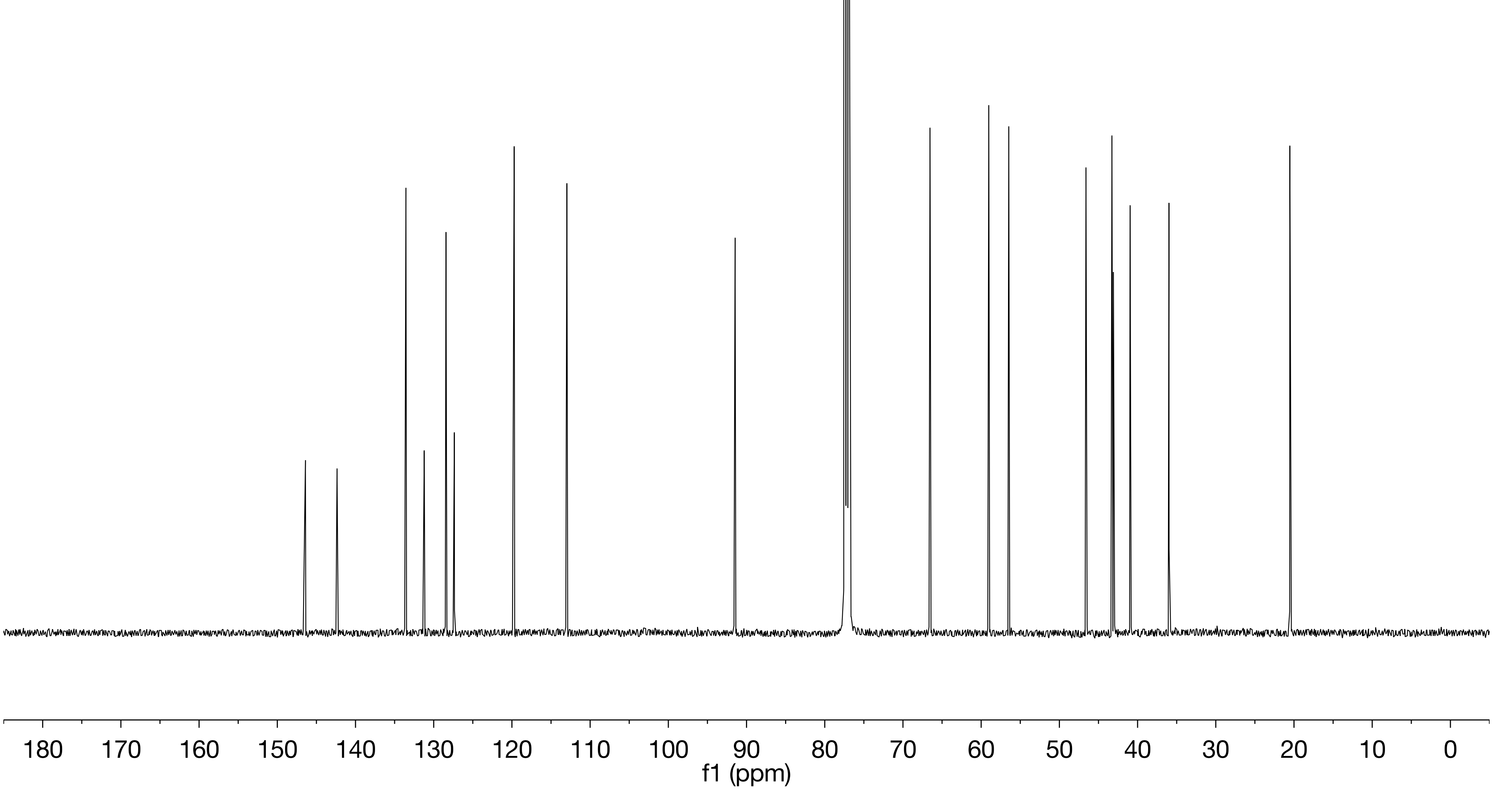

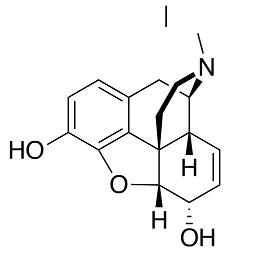

Morphine
^{1}H NMR, $CDCl_3$, 500 MHz

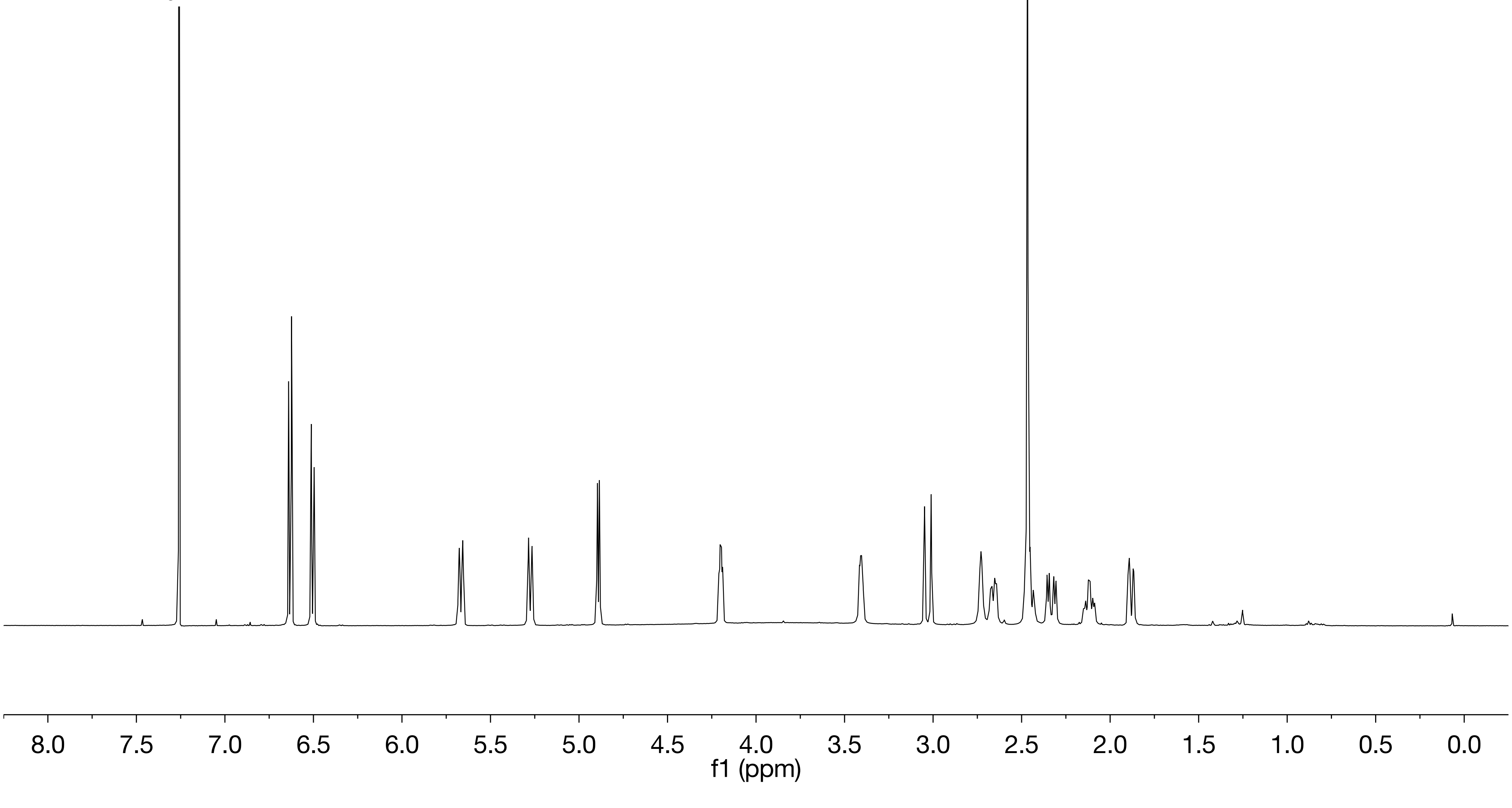

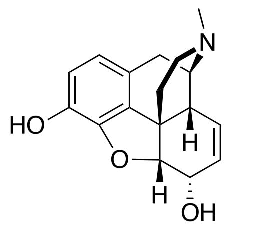

Morphine
^{13}C NMR, $CDCl_3$, 125 MHz

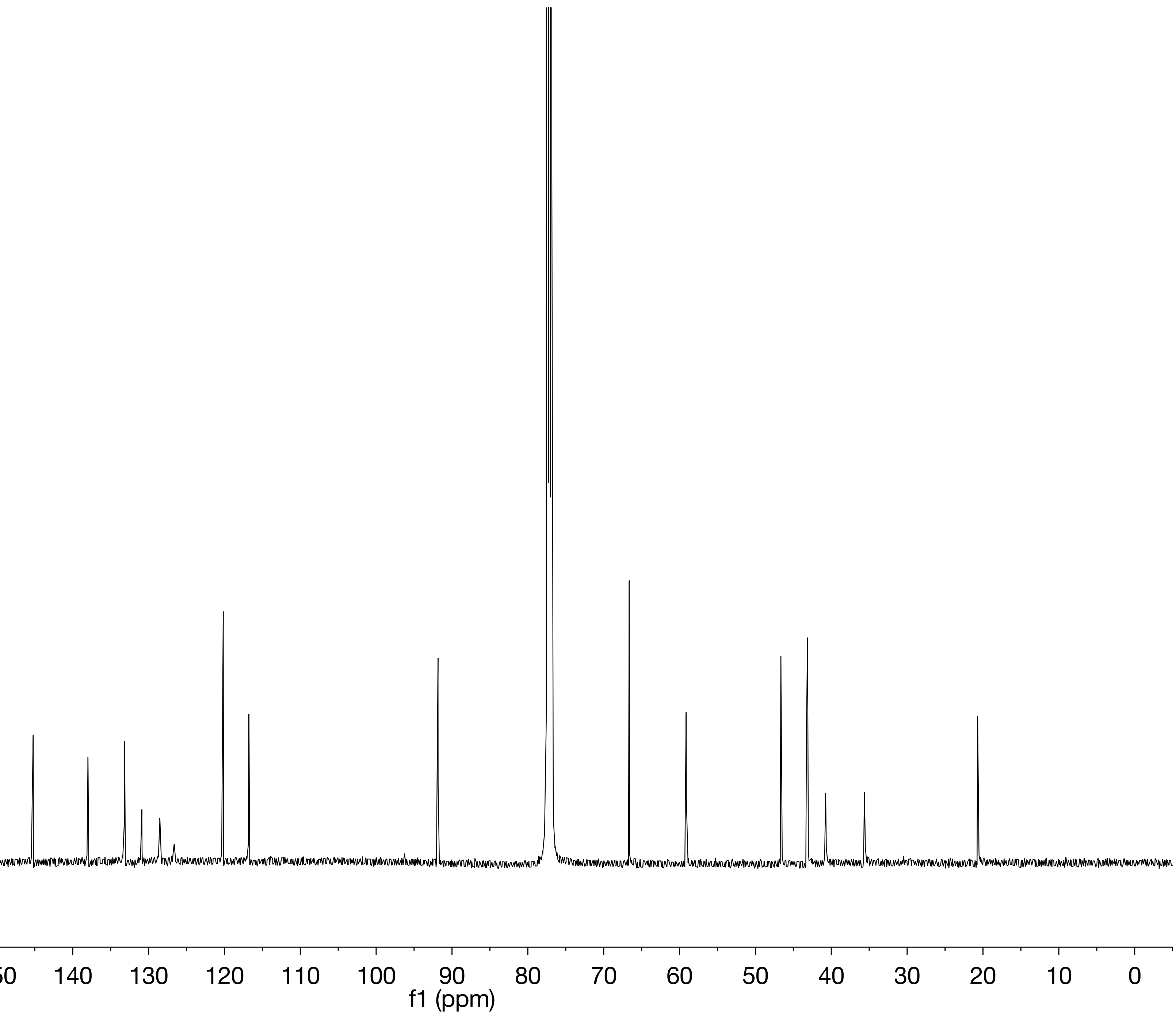

Supplement: Supplementary file 1 — Supplementary [file ANIE-55-14306-s001.pdf]
